# Brown Seaweed Food Supplementation: Effects on Allergy and Inflammation and Its Consequences

**DOI:** 10.3390/nu13082613

**Published:** 2021-07-29

**Authors:** Simone E. M. Olsthoorn, Xi Wang, Berend Tillema, Tim Vanmierlo, Stefan Kraan, Pieter J. M. Leenen, Monique T. Mulder

**Affiliations:** 1 Laboratory of Vascular Medicine, Department of Internal Medicine, Erasmus MC, University Medical Center, 3015 GD Rotterdam, The Netherlands; olsthoorn98@gmail.com (S.E.M.O.); wangxihnzyu@gmail.com (X.W.); 2 Department of Immunology, Erasmus MC, University Medical Center, 3015 GD Rotterdam, The Netherlands; p.leenen@erasmusmc.nl; 3 The Basic Medical Laboratory, Hunan University of Chinese Medicine, Changsha 410208, China; 4 Seaweed Food Solutions BV, 8444 DC Heerenveen, The Netherlands; tillema@boxnv.nl; 5 Neuroimmune Connections & Repair (NIC&R), Biomedical Research Institute (Biomed), Hasselt University, B-3590 Diepenbeek, Belgium; tim.vanmierlo@uhasselt.be; 6 Department of Immunology and Biochemistry, Biomedical Research Institute (Biomed), Hasselt University, B-3590 Diepenbeek, Belgium; 7 R&D Unit, Carnmore, Oranmore, H91 E09X Galway, Ireland; stefan.kraan@theseaweedcompany.com; 8 The Seaweed Company Blue Turtle, Co., Ltd., H91 E09X Galway, Ireland

**Keywords:** seaweed, allergy, inflammation, oral

## Abstract

Multiple health benefits have been ascribed to brown seaweeds that are used traditionally as dietary component mostly in Asia. This systematic review summarizes information on the impact of brown seaweeds or components on inflammation, and inflammation-related pathologies, such as allergies, diabetes mellitus and obesity. We focus on oral supplementation thus intending the use of brown seaweeds as food additives. Despite the great diversity of experimental systems in which distinct species and compounds were tested for their effects on inflammation and immunity, a remarkably homogeneous picture arises. The predominant effects of consumption of brown seaweeds or compounds can be classified into three categories: (1) inhibition of reactive oxygen species, known to be important drivers of inflammation; (2) regulation, i.e., in most cases inhibition of proinflammatory NF-κB signaling; (3) modulation of adaptive immune responses, in particular by interfering with T-helper cell polarization. Over the last decades, several inflammation-related diseases have increased substantially. These include allergies and autoimmune diseases as well as morbidities associated with lifestyle and aging. In this light, further development of brown seaweeds and seaweed compounds as functional foods and nutriceuticals might contribute to combat these challenges.

## 1. Introduction

Brown algae are one of three types of algae, i.e., brown algae (Phaeophyta), red algae (Rhodophyta) and green algae (Chlorophyta) classified based on their color and major photosynthetic pigments. Brown algae contain chlorophyll a, chlorophyll c and fucoxanthin, red algae contain chlorophyll a, chlorophyll d and phycoerythrin, while green algae contain chlorophyll a, chlorophyll b and xanthophylls. Due to the different abiotic and biotic factors in the marine environment and their distinct evolutionary origin seaweeds are a rich source of unique compounds of which several have demonstrated health benefits. Bioactive compounds of interest found in brown seaweed include polysaccharides (e.g., alginate, fucoidan), proteins (e.g., phycobiliproteins), polyphenols (e.g., phlorotannins), carotenoids (e.g., fucoxanthin), phytosterols (fucosterol) and *n*-3 long-chain polyunsaturated fatty acids (e.g., eicosapentaenoic acid) [1]. They have been reported to have beneficial effects in various diseases, including metabolic diseases, diabetes mellitus, cardiovascular disease, cancer and neurodegenerative diseases.

We structure this review according to the distinct compounds in brown seaweed that have been applied, and therefore distinguish effects of whole seaweeds or crude extracts (described in Section 3), polysaccharides such as fucoidan and alginate (Section 4), compounds with ring-shaped structures such as phytosterols (e.g., fucosterol) and (poly)phenols (e.g., phloroglucinol and phlorotannins) (Section 5) and carotenoids, in particular fucoxanthin, fucoxanthinol and meroterpenoids (Section 6).

**Figure 1:**
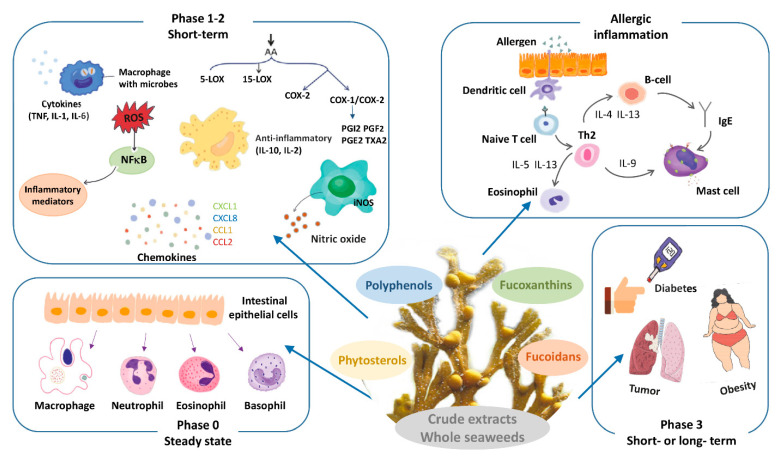
Schematic representation of brown seaweed and it constituents and of the phases in the immune response (Phase 0: steady state, allergy, Phase 1–2: acute inflammatory response and Phase 3: chronic inflammatory response) addressed in this review.

For each of these compounds we discuss the demonstrated effects on different phases of the inflammatory response (schematically depicted in Figure 1). We describe (i) studies that investigate the brown seaweed (compound) effects on steady state immune parameters that influence subsequent responses to inflammatory challenges. Then, in view of the focus of this special issue on allergic disease, we separately discuss (ii) studies aimed at identifying the effects of intake of brown seaweed or -components on allergies and models thereof. As next steps (iii) and (iv), we distinguish brown seaweed effects on different phases in other acute inflammatory responses, with their characteristic read-outs as indicated in Table 1. Briefly, here we distinguish the first, immediate response (iii) typified by reactive oxygen radical production and release of early mediators such as IL-1, TNF and arachidonic acid metabolites. Then, we consider (iv) the seaweed effects on the second inflammation phase characterized by enhanced production of above-mentioned cytokines as well as secondary cytokines, such as IL-10, chemokines and other mediators, and infiltration of leukocytes in tissue. Finally, we discuss (v) the effects of seaweed intake on chronic inflammation, which is mostly low-grade, and their sequels. These include insulin-, leptin- or glucocorticoid resistance, which are related to the induction of diabetes, obesity and hampered down-regulation of inflammation, respectively. Furthermore, malignant transformation may be another consequence of chronic exposure to inflammatory conditions.

Box 1 **Brief introduction on the inflammatory response.**

Inflammation is the response of tissues to any trigger significantly disturbing homeostatic conditions. These triggers can be manifold, and vary from infection or trauma, to metabolic challenges. The response is initiated by recognition of damage- or danger-related molecules (so-called DAMPs) that become exposed and are sensed by parenchymal tissue cells and resident innate immune cells, in particular macrophages and mast cells.

Activation of these cells (Phase 1) stimulates a cascade of events, within the first seconds to minutes, involving local release of ready-made mediators such as histamine, inflammatory arachidonic acid products such as PGE_2_, and stored cytokines. Oxygen radicals (reactive oxygen species; ROS), produced by NADPH-oxidase complex assembled upon cellular activation and by mitochondria, further amplify activation of resident cells. In addition to their essential role in redox signaling, ROS are important as microbicidal molecules and as inducers of oxidative damage. Secreted first wave mediators alarm neighboring cells and stimulate local vascular cell activation, causing upregulation of adhesion molecules on the endothelial cell surface, and vasodilation. This enables plasma fluid, including bioactive proteins, to penetrate the tissue.

Subsequently, activated resident cells produce inflammatory cytokines and chemokines by de novo gene transcription (Phase 2). Inflammatory signaling pathways involving NF-κB and AP-1, triggered by the receptor-DAMP interaction, are essential in this process. The released mediators amplify the inflammatory response and enable the recruitment of leukocytes from the circulation. Based on the profile of released chemokines, neutrophils are the most numerous attracted cells in the initial response. In addition, expression of inducible nitric oxide synthase (iNOS) is stimulated by bacterial products and pro-inflammatory cytokines, in particular IFN-γ. iNOS generates nitric oxide (NO), which is an important inflammatory mediator as microbicidal product of especially activated macrophages, and as an autocrine and paracrine signaling molecule. In this second phase of inflammation, also anti-inflammatory mediators are produced, which include arachidonic products such as lipoxins and resolvins, and cytokines such as IL-10. Dependent on the strength of the inflammatory trigger, systemic responses may occur, including activation of the hypothalamus-pituitary-adrenal (HPA) axis, leading to cortisol or corticosterone release. The first systemic cytokine wave initiates the production of so-called acute phase proteins such as C-reactive protein (CRP) from the liver, which occurs from approximately 24 h after the initial trigger. An adaptive immune response is initiated as DAMPs and -related antigens are transported to draining lymph nodes via lymph and migrating antigen-presenting cells. This leads to proliferation of antigen-specific B- and T-lymphocytes that eventually leave the lymph nodes and become effective in the periphery from approximately 4 days after initiation.

Dependent on the nature and severity of the initial trigger, and ability to annihilate it, repair processes are initiated after a few days. If the trigger remains, such as in case of adverse metabolic conditions or persistent infection, inflammation may become chronic. Then a new equilibrium is sought (Phase 3), which affects tissue performance and the response to further environmental triggers. Long-term exposure to adverse conditions thus may lead for instance to resistance to regulating hormones, such as insulin or cortisol.

Allergic responses are inflammatory responses triggered by pre-existing antibodies or primed T-lymphocytes specific for harmless molecules from the environment. Best known are the responses caused by activation of mast cells evoked by IgE antibodies specific for innocuous antigens such as house dust mite or pollen. Other antibody subclasses with adverse specificities, for instance against certain drugs, also can cause clinical responses. Finally, T-lymphocytes with unfavorable specificities may cause so-called delayed-type hypersensitivity responses. The delay is explained by the necessity of T-lymphocytes to migrate to the site of antigen exposure and presentation, and to become activated locally. In essence, allergic responses follow the phases as outlined for the inflammatory response in general.

Challenges to generating a comprehensible overview are the multifaceted aspects of allergy and inflammation, besides the large variety of brown seaweed species and preparations, as well as the different host species in which seaweed administration has been tested. Furthermore, this divergency bears a risk of overstretching conclusions based on limited findings. With these caveats in mind, we attempt to describe the commonalities between different studies, or discuss specific findings that deserve attention for future development. Detailed findings from the individual publications have been summarized in tables, linked to the compound categories mentioned above.

## 2. Approach to Systematic Search

The aim of this systematic review is to investigate the effect of brown seaweed components as a dietary supplement on inflammation with extra focus on allergies.

### 2.1. Databases and Search Strategy

We performed two searches, in May and in November 2020. Several electronic databases were used to include studies: Embase via Embase.com (1971–Present), Medline ALL via Ovid (1946–Present), Web of Science Core Collection via Web of Knowledge (1975–Present), Cochrane Central Register of Controlled Trials via Wiley (1992–Present) and Google Scholar. References were filtered for duplicates in Endnote. The systematic search was executed by Elise Krabbendam, Biomedical Information Specialist at the Medical Library of Erasmus MC. The exact search terms are shown in the Supplementary Materials, where also a detailed flow chart of paper selection is shown. First, at least two review authors independently assessed title and abstract of the articles based on inclusion and exclusion criteria mentioned below. This was carried out using Endnote X9 software, based on reviewing methods described by Bramer et al. 2017 [2]. Secondly, after independent assessment of the articles, references selected by both review authors were included in a preliminary database. For final full text inclusion all references were combined and assessed by all review authors to assure eligibility and to extract important information to be included in the tables. References were divided by seaweed component among all review authors and based on full text selected to be included or excluded from the systematic review. Third, some relevant articles were included that were not retrieved in the systematic search, primarily because searchable parts of the articles did not contain reference to terms related to inflammation.

### 2.2. Eligibility Criteria

For this systematic review we only included articles written in English exclusively. Review articles were excluded from the search. The aim of the first search was to include papers addressing the effect of brown seaweed as nutritional supplement on leucocytes and how this contributes to the inflammatory process. This resulted in a total of 906 references after deduplication. For extra focus on allergy and atopy the search was expanded, resulting in a final total of 1035 references after deduplication. Papers on components of brown seaweed or whole extracts were included, but only when orally administered to human or animals. Exclusion criteria for this systematic review were seaweeds other than brown species or no oral administered. Furthermore, articles mentioning fucoidan as control inhibitor/blocker for selectins or scavenger receptor exclusively, were also excluded.

## 3. Whole Seaweed or Crude Extract Supplementation

### 3.1. Whole Seaweed or Crude Extract: Effects in Steady State

Table A1 summarizes the main findings of oral supplementation of whole seaweeds or crude extracts on inflammation-related parameters. Safety aspects of seaweed consumption are important to consider. Potential overload with iodine or heavy metals are major long-term risks when unprocessed edible seaweeds are ingested [1]. Short-term monitoring after administration of single doses of crude extracts in rodents showed no effects of toxicity up to 5000 mg/kg in mice (*Sargassum micracanthum* [3]; *Cystoseira compressa* (Esper) [4]), or showed LD50 values of 1000–2000 mg/kg (*Fucus vesiculosis* [5]). Considering a dosage of 200 mg/kg/day is routinely applied for regular use, it may be argued that the safe dosage range might be limited. However, specific toxicity may be highly batch-dependent and related to toxic contaminants rather than seaweed content.

A widely studied facet of seaweed supplementation is its high anti-oxidant activity, and related to this, anti-inflammatory activity. Human studies in this direction are scarce, however. Consumption of 4.8 g dried *Sargassum muticum* per day for a period of 4 weeks by healthy volunteers stimulated an increased total anti-oxidant status in serum, correlated to decreased concentrations of oxidized LDL [6]. In contrast to general assumptions, Baldrick et al. observed no significant changes in oxidative or inflammatory parameters after oral consumption of *Ascophyllum nodosum* extract by individuals with overweight or obesity (100 mg/day, 8 weeks) [7]. An interesting aspect of the latter study is that individuals differed notably (up to >4000×) in the total amount of seaweed polyphenol metabolites present in urine. It is tempting to speculate this might be attributed to differences in microbiota composition between individuals. In a study in goat, where *Ascophyllum nodosum* extract was added (2%) to feed, an increased anti-oxidant status was shown [8]. Similarly, 4-week treatment of rats with *Fucus vesiculosus* extract stimulated increased serum paraoxonase and superoxide dismutase activities, thus leading to an increased anti-oxidant status [5].

Effects of seaweed supplementation on blood cell counts are variable; studies in human or other mammals showed limited effects [6,8], but increased counts were observed when chicken and fish were fed *Laminaria japonica* or *Sargassum oligocystum*, respectively [9,10]. Such addition to animal feed is not only associated with increased growth and feed conversion ratios in chicken and fish, but also enhanced status of innate and adaptive immune defenses and immune responsiveness and survival after infectious challenge [11,12,13].

### 3.2. Whole Seaweed or Crude Extract: Effects on Allergy 

Importantly, oral brown seaweed supplementation shows consistent beneficial effects in different models of allergy (Table A1). *Eisenia* (=*Ecklonia*) *arborea* powder intake by Brown Norway rats, orally sensitized to ovalbumin, leads to decreased serum IgE and histamine levels and decreased IL-4 and IL-10 production in lymphoid organs, while IFN-γ synthesis is increased. This indicates a favorable change in Th1/Th2 balance towards the former [14].

Mouse models using 2,4-dinitrophenol (DNP) or 2,4-dinitrochlorobenzene (DNCB) sensibilization and challenge are much used in this field, and similarly show positive effects of brown seaweed intake. *Sargassum horneri* extract has anti-allergic activity by suppressing degranulation of mast cells and basophils. This reduces nasal rubbing [15] or clinical signs of atopic dermatitis, as well as inflammatory cytokine levels and leukocyte skin infiltrates [16] in these allergy models. Similarly, application of extracts from *Costaria costata* [17] and *Laminaria japonica* [18] reduce severity of allergic dermatitis and stimulate healing, possibly by decreasing inflammatory pathways in keratinocytes.

### 3.3. Whole Seaweed or Crude Extract: Effects in Acute and Chronic Inflammation

Without prior sensitization, irritant application on murine skin also causes signs of acute inflammation associated with local mast cell degranulation and increased vascular permeability. Feeding rats with *Laminaria japonica* extract decreases paw swelling and leukocyte infiltration induced by carrageenan application, likely by inhibiting NF-κB activation causing decreased inflammatory mediator production [19] (Table A1). Similarly, oral or topical administration of *Sargassum fusiforme* extract significantly reduces ear swelling by inhibiting mast cell degranulation and enzymes involved in production of inflammatory arachidonic acid mediators [20].

The challenge of experimental animals or isolated cells with lipopolysaccharide (LPS) is a common model of acute inflammation, mimicking the response to bacterial infection. A 5-week treatment of rats with *Eisenia* (=*Ecklonia*) *bicyclis* extract mediated a reduced inflammatory activation of peritoneal macrophages upon in vitro LPS stimulation through inhibition of NF-κB activity [21]. This was associated with reduced iNOS expression and nitric oxide (NO) production. In marked contrast, a similar study in mice showed that 3-week oral treatment with *Sargassum fusiforme* (also called *Hizikia*) extract slightly potentiated the production of IL-1β, IL-6 and TNF-α by isolated peritoneal macrophages stimulated in vitro with LPS [22]. In accordance with an inflammation-regulating effect, 4-day oral application of *Sargassum serratifolium* extract in mice caused significantly reduced production of TNF-α, IL-1β and IL-6 upon in vivo LPS challenge [23]. This confirmed the in vitro findings of direct inhibition of NF-κB activation and nuclear translocation by seaweed components.

Investigation of seaweed treatment on LPS responses in other than murine species generally corroborated an inflammation-inhibiting effect. Challenge of zebrafish embryos with LPS or H_2_O_2_ showed reduced reactive oxygen species (ROS) production and associated cell death when treated simultaneously with *Sargassum polycystum* or *Chnoospora minima* extract [24].

Adding *Sargassum latifolium* to sheep feed caused a reduced inflammatory response to LPS challenge and increased blood anti-oxidant defense capacity [25]. This seaweed treatment also mediated a reduced inflammatory response to heat stress challenge in these sheep [26]. The latter study confirms earlier work in lamb, showing reduction of heat stress-related effects on leukocyte oxidative and phagocytic function by *Ascophyllum nodosum* extract administration [27].

Furthermore, in mouse models of inflammatory disease, seaweed supplementation has shown beneficial effects. In dextran sulfate sodium-induced chronic colitis, application of *Turbinaria ornata* extract causes decreased disease activity as indicated by colon length, histomorphological index and myeloperoxidase activity [28]. This was accompanied by increased expression of regulatory T-cell-associated FoxP3 and anti-inflammatory IL-10. Similarly, *Laminaria japonica* extract caused a significant diminution of colitis signs in this model, and simultaneous application of bacterial probiotics showed synergistic beneficial effects on histological score and decreased levels of some proinflammatory cytokines [29]. In a mouse model of arthritis induced by bovine type II collagen immunization, oral supplementation with *Sargassum muticum* extract significantly decreased the arthritis and edema scores as well as TNF, IL-6 and IFN-γ levels [30].

In one of the scarce human studies, Cooper et al. found that individuals with active Herpes infection showed increased healing rates with *Undaria pinnatifida* consumption, while latent Herpes carriers did not experience viral reactivation [31]. Investigating the mechanisms, the authors found the extract strongly inhibited Herpes virus infectivity in vitro, and stimulated human T cell mitogenesis, thus potentiating adaptive immune responses.

A study using a phylogenetically more distinct organism, kuruma shrimp, indicated that oral supplementation with *Laminaria japonica* significantly increased survival upon White Spot Syndrome virus infection [32]. This was accompanied by enhancement of chemotaxis as well as other defense mechanisms by hemolymph leukocytes (hemocytes), including superoxide production and antioxidative phenoloxidase activity upon appropriate stimulation.

### 3.4. Whole Seaweed or Crude Extract: Late Consequences of Inflammation and Sequels 

Acute or chronic inflammatory conditions influence local and systemic tissue responses, and thus seaweed supplementation also affects peripheral tissue function in inflammation (Table A1). In a rat model of ligature-induced periodontitis, *Sargassum fusiforme (Hizikia)* application reduced alveolar bone loss, related to decreased osteoclast and increased osteoblast gene expression in vitro [33]. Furthermore, in a model of autoimmune thyroiditis, a traditional Chinese medicine combination of 10 different herbs, including *Sargassum fusiforme*, mediated a decrease in autoimmune thyroiditis and anti-thyroid autoantibody formation [34]. Omission of *Sargassum fusiforme* in this model significantly diminished the protective effect.

In recent years, the link between adipose tissue metabolic dysregulation and inflammation has been recognized increasingly [35]. Several studies investigated metabolic effects of seaweed application, in particular *Undaria pinnatifida*, in murine models of obesity and type 2 diabetes induced by a high-fat diet [36,37,38]. Seaweed was in some studies combined with other nutraceuticals. In general, the obesity phenotype did not change, while improvement of glucose regulation was only observed by Oh et al. (2016) [36], but not in the other two studies. Other aspects, however, showed beneficial effects of seaweed supplementation, such as microbiome composition, MCP-1 induction [38], systolic blood pressure and non-esterified fatty acid levels [37] or presence of clusters of necrotic adipocytes surrounded by macrophages in adipose tissue (so-called crown-like structures) [36].

A pathological condition strongly related to obesity is the polycystic ovary syndrome (PCOS). In a rat model of PCOS, application of *Ecklonia cava* extract mediated a decrease in vaginal leukocyte infiltration, and restored hormonal levels and irregular ovarian cycles [39]. However, it did not inhibit the weight gain associated with PCOS induction.

## 4. Brown Seaweed Polysaccharide Supplementation 

Among polysaccharides present in brown seaweed fucoidan has received most attention as a constituent with diverse bioactive effects. Furthermore, bioactivity has been demonstrated of the polysaccharides laminarin, a beta-glucan polysaccharide and alginate, a linear acidic soluble dietary polysaccharide.

Fucoidans are a group of polysaccharides (fucans) primarily composed of sulphated L-fucose with less than 10% of other monosaccharides. They are widely found in the cell walls of brown seaweeds, but not in other algae or higher terrestrial plants [40]. The major function of fucoidans in cell walls is mechanical support and protection against desiccation during air-exposure of the seaweed at low tide. The amount of fucoidan in brown seaweeds is variable; 8–20% of dry weight with the highest content of about 20% being detected in *Fucus* *vesiculosus* [41,42].

A number of health-improving effects have been ascribed to fucoidans [40,41,43]. Biological activities of fucoidans, such as antioxidant and anti-coagulant capacity, are affected by their molecular weight and sulphated ester content, both the number of sulphate groups, determining the negative charge of the molecule and the position of the sulphate groups on the sugar residues [40,44]. The biological activity of fucoidan is also affected by the glucuronic acid and fucose content. The molecular weight of fucoidan ranges from for example from 50 to 80 KDa in *Undaria* *pinnatifida* and *Fucus* *vesiculosus,* respectively, to 1920 kDa in *Cladosiphon* species [45], with multiple sizes being present in certain species. Low molecular weight (LMW) fucoidan is produced by enzymatic digestion or acid hydrolysis of naturally occurring high molecular weight (HWM) fucoidan. Application of different molecular species of fucoidan obtained by different methods of purification and treatments such as hydrolysis complicates interpretation of results.

### 4.1. Brown Seaweed Polysaccharide Effects in Steady State

Fucoidan is absorbed in limited amounts in the gastrointestinal tract after oral intake [46,47]. In Japanese populations where brown seaweed is part of daily diet, systemic fucoidan uptake was shown by its presence in serum and urine [48]. Protective effects of fucoidan on the intestinal epithelial barrier function were observed in vitro. Fucoidan protected the tight junctions from oxidative injury and upregulated the expression of claudin-1 [49]. Table A2 summarizes the effects of fucoidan on different aspects of inflammation.

Fucoidan is not toxic, but high dosages can induce increased bleeding time. In rats no toxicity as observed after oral administration of a single dose of *Ascophyllum nodosum* fucoidan of 2000 mg/kg [50] or 300 mg/kg/day *Laminaria japonica* fucoidan for 6 months [51]. However, application of a daily dose of 2500 mg/kg for 6 months resulted in increased bleeding time. The application of fucoidan in food has been approved for human consumption up to 250 mg/day by the European Food Safety Authority, EFSA [1].

### 4.2. Effects of Polysaccharides from Brown Seaweed in Allergy, Acute Inflammation and in the Modulation of Immune Responses

Both pro- and anti-inflammatory effects of fucoidan have been reported. In macrophages fucoidan treatment induced NF-κB nuclear translocation, followed by iNOS and COX-2 transcription, inducing the secretion of the pro-inflammatory cytokines IFN-γ, TNF-α and IL-1β and of inflammatory mediators NO and PGE2 [52]. However, pre-treatment of macrophages and lymphocytes with fucoidan prior to stimulation with LPS or other pro-inflammatory stimuli was found to blunt the pro-inflammatory reaction or induces an anti-inflammatory effect, resulting in inhibition of NF-κB translocation and in lower levels of pro-inflammatory mediator production [53,54,55,56].

Below an elaboration on anti-allergy effects and enhanced immune effects in production animals and innate and adaptive immune system modulation studies in mice is described.

#### 4.2.1. Anti-Allergic Effects of Brown Seaweed-Derived Polysaccharides

Overall, oral supplementation of brown seaweed polysaccharides was reported to inhibit allergic responses via multiple mechanisms. The polysaccharides were shown to be an effective agent antagonizing IgE production as examined in different ovalbumin (OVA)-sensitized mouse models [57,58], but also an allergy-specific mechanism of oral fucoidan supplementation has been found in its capacity to induce galectin-9 production from intestinal cells [59,60]. Galectin-9, belonging to a soluble lectin family, recognizes β-galactoside and prevents IgE binding to mast cells, consequently inhibiting mast cell degranulation. Accordingly, fucoidan from *Saccharina japonica* (400 μg for 4 days) was found to increase circulating galectin-9 [59]. After OVA-immunization the allergic symptoms in sensitized mice were reduced by fucoidan (60 μg/mouse/d for 17 days) via inducing galectin-9 production from colonic epithelial cells [60].

In several OVA-immunized mouse models, oral administration of fucoidan or a polysaccharide fraction was shown to have anti-allergy activity. Application of a polysaccharide fraction from *Laminaria japonica* (50 mg/kg/day for 2 weeks) in a mouse model of asthma significantly decreased the numbers of eosinophils in the bronchoalveolar fluid and alleviated lung inflammation compared to the non-treated control mice [58]. It also reduced serum IgE concentrations and decreased the concentrations of IL-13 and TGF-β1 in bronchoalveolar fluid and expression in lung, while increasing expression of IL-12. Similarly, *Laminaria japonica* fucoidan ingestion (200, 600, 1000 mg/kg for 6 weeks) decreased OVA-specific IgE in mice [61]. Fucoidan from *Undaria* *pinnatifida* (400 mg/kg for 7 days) inhibited particulate matter-induced exacerbation of allergic asthma [57]. Specifically, fucoidan treatment significantly attenuated lipid peroxidation, infiltration of inflammatory cells and Th2-related IL-4 concentrations. Furthermore, fucoidan suppressed mast cell activation, degranulation and IgE synthesis as well as mucus hypersecretion and goblet cell hyperplasia. This also is reflected in immunoglobulin isotypes produced as *Cladosiphon*-fucoidan dose-dependently (up to 1025 mg/kg body weight for 8 weeks) increased systemic IgM, IgG and IgA levels, while decreasing IgE and IL-4 significantly [62].

The observed changes are suggestive of a shift from Th2 to Th1 induced by orally ingested fucoidan. Enhanced IL-12 and IFN-γ production by ingestion of *Tetragenococcus halophilus* KK221, a probiotic known for its anti-allergic properties, was even further increased by combined ingestion of the probiotic and LMW fucoidan isolated from *Undaria pinnatifida* in OVA-immunized mice. The results indicated an extra shift towards Th1.

Furthermore, alginate was found to improve (food) allergy outcomes in an OVA-sensitized mouse model [63]. Ingestion of alginate (2 mg) extracted from *Laminaria japonica* one day before oral application of ovalbumin improved integrity of intestinal epithelial villi and inhibited mast cell degranulation in the jejunum. Serum levels of IgE, histamine and IL-4 were significantly lower, while IFN-γ was markedly increased. Furthermore, Tregs in spleen were increased, while OVA-induced differentiation of Th0 cells into Th2 cells was inhibited [63].

Overall, brown seaweed-derived polysaccharides generally appear to modulate the Th1/Th2 balance and mast cell degranulation in favor of an anti-allergic effect. This shows that fucoidan is potentially an effective therapeutic agent for type I allergic diseases.

#### 4.2.2. Effects of Brown Seaweed Polysaccharides on Innate and Adaptive Immune System (Production Animals)

In search for alternatives for antibiotics in production animals, brown seaweed polysaccharides and especially fucoidan, have appeared as promising functional feed additives. Fucoidan and laminarin were found to improve the immune response of pregnant sows and piglets prior to or while suckling [64], and after weaning [65].

Dietary supplementation of sows in the final part of gestation with *Laminaria* spp. extract increased IgG and IgA in sow colostrum by 19% to 25% [64]. Consequently, also a 10% increase in piglet serum IgG was observed. This suggests an important effect of maternal diet on the immune status of piglets. Dietary supplementation with an extract of *Ascophyllum nodosum* and *Fucus* in sows (30 g/day from the 85th day of gestation until weaning) resulted in an increased population of CD4+CD8+ T cells in the thymus, spleen, mesenteric node, liver and in peripheral blood as compared to the control group [66]. Piglets from laminarin-fed sows (1.0 g/d from day 107 of gestation until weaning) showed down-regulation of IL-6 mRNA expression in the colon at weaning and of IL-8 in the ileum on day 8 post weaning compared with those from the non-laminarin-fed sows [67].

Weaning of piglets is a stressful event for piglets and is often associated with pro-inflammatory immune effects in the piglets’ gastro-intestinal tract. Addition of laminarin to weaning piglets’ diets resulted in lower expression of pro-inflammatory cytokines IL-1β, IL-6 and IL-17 in colonic mucosa [65]. Even though these positive effects were observed in piglets, laminarin did not result in any detectable benefits in Friesian bull calves [68].

In Salmonella-challenged broiler chickens, addition of 0.2% alginate oligosaccharides to the regular diet inhibited *Salmonella* *enteritis* colonization, possibly by increasing colonic anti-Salmonella IgA levels [69]. In unchallenged broiler chickens, supplementation of 0.2% alginate oligosaccharides showed dramatic immunostimulatory activity by inducing interferon-γ, IL-10 and IL-1β mRNA expression in cecal tonsils. Interestingly, the robust mucosal immune response in the absence of a challenge was related to a decline in body weight, as compared to the control group.

In aquaculture, several studies pointed at improved innate immune markers upon fucoidan and laminarin supplementation in shrimp and fish [70,71,72,73,74,75,76]. In addition, higher survival rates during bacterial challenges were observed in the supplemented animals, as compared with those fed a regular diet.

Taken together, enhancing the innate and adaptive immune system by oral ingestion of seaweed-derived polysaccharides and oligosaccharides is a promising solution for improving animal health, reducing infection incidence and reducing the need for antibiotics use.

#### 4.2.3. Effects of Brown Seaweed Polysaccharides on Innate and Adaptive Immune System (Mouse Models)

Polysaccharides obtained from brown seaweed may support various aspects of the immune system in both immunocompetent and immunosuppressed states. For instance, oral ingestion of a polysaccharide extract from *Kjellmaniella crassifolia* (2 weeks) by C57BL/6 mice, resulted in enhanced IFN-γ, IL-12, IL-6 and IgA secretions by spleen cell cultures upon concanavalin-A stimulation [77]. Orally administered LMW fucoidan (200–1000 mg/kg for 6 weeks) from *Laminaria japonica* to BALB/c mice stimulated the innate immune system by increasing natural killer (NK) cell activity and peritoneal macrophage phagocytic activity [61]. LMW fucoidan also increased IL-2, IL-4 and IFN-γ secretion by splenocytes and IgG and IgA concentrations in serum, while it decreased OVA-specific IgE. In bacterial antigen-stimulated immune responses, the IgM and IgG concentrations in serum were significantly higher in the LMW fucoidan group than in the control group. In addition, an LMW fucoidan-enriched extract from *Okinawa mozuku* orally administered (up to 1025.0 mg/kg for 6 weeks) to BALB/c mice resulted in enhanced splenocyte proliferation and secreted IL-2 levels, as well as in increased macrophage phagocytic activity, and serum IgM, IgG and IgA, while splenocyte-secreted IL-4 and IL-5 were decreased, and also serum IgE was decreased significantly [62]. Interestingly, HMW fucoidan (50 g/kg) but not LMW and IMW fucoidan, increased the relative number of cytotoxic T-cells in spleens of Balb/c mice [78]. These immune-potentiating effects appear to be effective in infection as complete elimination of liver and spleen parasite burden was achieved by fucoidan (200 mg/kg, 3 times weekly, for 6-weeks) in a mouse model of *Leishmania donovanii* infection [79]. This curative effect was associated with switching of T cell differentiation from Th2 to Th1 mode.

In addition to its capacity to enhance the innate and adaptive immune system, oral fucoidan is an interesting candidate for antiviral therapies related to its intrinsic capacity as a competitive binding agent for envelope viruses, thus preventing cellular entrance [80]. Oral ingestion of fucoidan improves the outcome in virus-infection mouse models with respect to viral load [81], serum antibody levels and overall survival [82,83] in immunocompetent and immune-suppressed mice. Furthermore, fucoidan extracted from *Undaria pinnatifida* protected both immunocompetent and immunosuppressed mice from infection with HSV-1 as indicated by the improved survival rate and lesion scores [82]. In immunocompetent mice fucoidan enhanced activity of CTL and increased circulating anti-HSV antibodies in HSV-1-infected mice.

In an immunosuppressed state, selective augmentation of NK activity was observed upon oral treatment with *Undaria* fucoidan, but this induced no significant change in NK activity in immunocompetent mice where a normal level of NK activity was maintained. Fucoidan extracted from *Undaria pinnatifida* showed also beneficial effects during influenza virus infection in immunocompetent an immunosuppressed mice [83]. Fucoidan administration (7 days prior to virus inoculation until 7 days after inoculation (2 × 5 mg/day)) resulted in significant increase in neutralizing antibody titers in bronchoalveolar lavage fluids in both healthy mice and mice with suppressed immunity as compared with placebo groups.

Furthermore, in the defense against tumors fucoidans enhance innate and adaptive immune responses. Different fucoidans were found to increase immune reactions in various tumor models, leading to comparable or even better results than standard chemotherapy exclusively [84,85,86,87]. Consumption of fucoidan isolated from *Undaria pinnatifida* (1% of the diet for a period of 10 days) showed tumor inhibition in an A20 leukemia mouse model, related to enhanced Th1 and NK cell activity [84]. Oral intake of polysaccharides from *Sargassum fusiforme* (100 and 200 mg/kg for 28 days) significantly inhibited the growth of A549 lung adenocarcinoma in mice, but also remarkably promoted IL-1 and TNF-α production from peritoneal macrophages, increased serum TNF-α levels and splenocyte proliferation [87]. Oral administration of fucoidan extracted from *Cladosiphon okamuranus* (5 g/kg/day for 28 days) also inhibited tumor growth and increased survival time in a colon 26 tumor-bearing mouse model. In the spleens of these mice, an increased population of NK cells was observed. Furthermore, in an experiment applying the same fucoidan to MyD88 knockout mice, a model for investigating TLR4 signaling pathways, it was found that the observed anti-tumor effects are related to gut immunity [85]. Furthermore, polysaccharide extract from *Sargassum fusiforme* (400 mg/kg for 28 days) exerted anti-tumor and immunomodulatory activities in nasopharyngeal carcinoma [86] and hepatic carcinoma tumor-bearing mice [88]. In a xenograft tumor model orally administered fucoidan from *Fucus vesiculosus* (150 mg/kg body weight daily for 2 weeks) increased cytolytic activity of NK cells and significantly delayed tumor growth [89]. Furthermore, in a rat model for experimental mammary carcinogenesis administration of fucoidan (400 mg/kg/day for 4 months) showed protective and immunomodulatory effects [90]. Tumor growth in Sarcoma 180 (S-180)-bearing mice was delayed by ingestion of fucoidan from *Cladosiphon okamuranus*, which stimulated NO production by macrophages via NF-κB-dependent signaling pathways [52]. Oral intake of ascophyllan, a sulphated polysaccharide obtained from *Ascophyllum nodosum,* (50 and 500 mg/kg), also delayed tumor growth. Interestingly, oral ingestion significantly increased serum IL-12 and TNF-α levels and mediated better overall outcome compared to intraperitoneal application in S-180 mice, where immune markers did not change [91].

Seaweed polysaccharides can also function as immune-stimulating adjuvant in immunosuppressed states during chemotherapy. Oral intake of polysaccharide extract from *Sargassum fusiforme* (200 mg/kg for 6 days) was identified as a potent immune-enhancing agent in immunosuppressed mice [92]. Oral administration of fucoidan (150 mg/kg for 14 days) resulted in enhanced recovery of all T cell populations (CD3+, CD4+, CD8+) and of the proliferative capacity of splenocytes in immunosuppressed mice [93]. Furthermore, laminarin administration (500–1000 mg/kg/day for 10 days) induced IL-12 and IFN-γ in immunosuppressed mice [94]. Taken together, oral intake of brown seaweed polysaccharides is shown to be an effective immune enhancer in a wide variety of mouse models.

#### 4.2.4. Anti-Inflammatory Effects of Fucoidan in Animal Models and Clinical Trials

Fucoidans extracted from different seaweed species and molecular sizes showed anti-inflammatory effect in a wide range of acute and chronic inflammation models in mice.

In an arachidonic acid-induced ear inflammation model sulphated polysaccharide extracted from *Sargassum* *hemiphyllum* decreased ear swelling and erythema [95]. The polysaccharides decreased the local levels of myeloperoxidase, nitric oxide, IL-1β, IL-6 and TNF-α in a dose-dependent manner (20–80 mg/kg body weight for 5 consecutive days). Histological examination revealed that the polysaccharides reduced the area of neutrophilic infiltration in inflamed ears. Similarly, oral ingestion of fucoidan extracted from *Undaria* *pinnatifida* (0.5 mg for 20 days) inhibited the inflammatory reaction in a mouse model where LPS was injected buccally [55]. In the same set up, but now using bacterial infection, fucoidan reduced inflammation but did not lead to clearance of the bacterial infection, nor to prevention of infection-related bone loss. In a carrageenan-induced air pouch inflammation model, a preparation of fucoidan (54 mg/kg for 7 days) inhibited inflammatory markers and showed reduced attraction of inflammatory cells as demonstrated by histology [96]. Pretreatment with orally administered fucoidan (20 mg/kg for 2 weeks) reduced mucosal lining inflammation and prevented elevation of serum IL-6 levels, while levels of serum IL-10 increased in an aspirin-induced mucosal ulcer model in mice [97]. Accordingly, *Cladosiphon* fucoidan (chow containing 0.05% *w*/*w*) ingestion beneficially affected murine dextran sulphate sodium (DSS)-induced colitis [98]. The lamina propria of inflamed colon showed reduced amounts of IL-6 and IFN-γ, and an increase in IL-10 and TGF-β upon fucoidan treatment. Murine DSS-induced colitis significantly improved upon treatment with fucoidan (10 mg/day for 1 week) [99]. Treatment with a fucoidan-polyphenol complex showed even better results as it reduced IL-12, TNF-α and IL-6 in colitis tissue and ameliorated colitis-related visible body markers, such as weight loss and blood in stool. In contrast, when this complex was injected intraperitoneally, it was unable to reduce disease severity and even deteriorated some colitis markers. In mice, colonic inflammation and microbiota dysbiosis induced by antibiotics was alleviated by administration of fucoidan extracted from *Ascophyllum nodosum* (400 mg/kg for 28 days) [100]. Fucoidan prevented colon shortening and colon tissue damage, and it improved abundance of beneficial microbes while decreasing harmful microbes. In a model with chemically induced colorectal cancer, ingestion of *Fucus* *vesiculosus* fucoidan was shown to protect against tumorigenesis and to reduce colorectal inflammation and dysbiosis [101].

The bioactivity of fucoidan is related to its molecular weight. LMW and HMW fucoidan from *Undaria pinnatifada* were tested in a murine model of collagen-induced arthritis [102]. LMW fucoidan protected against tissue degeneration, while the same dose of HMW fucoidan worsened it. In accordance, LMWF reduced the severity of arthritis and the levels of Th1-dependent collagen-specific IgG2a, while HMWF enhanced the severity arthritis and the levels of collagen-specific antibodies.

Furthermore, in different acute inflammation models in zebrafish embryos, strikingly similar anti-inflammatory effects were noticed [53,54,56,103]. Administration of fucoidan (25–100 µg/mL) one hour prior to LPS treatment improved survival of zebrafish embryos and diminished inflammatory markers.

In support of an inflammation-regulating effect of fucoidan, oral administration of *Laminaria japonica* fucoidan (50–200 mg/kg) protected against myocardial ischemia–reperfusion injury in rats in a dose-dependent manner [104]. Furthermore, oral ingestion of enzymatically hydrolyzed fucoidan extracted from *Sargassum* *hemiphyllum* (200 mg/kg/day for 14 days) decreased radiation-induced pneumonitis and lung fibrosis by reducing inflammatory cytokine expression in lung tissues [105]. In both models decreased accumulation of neutrophils and macrophages was observed.

In a mouse model of chronic infection with *Schistosoma japonicum* oral ingestion of *Fucus vesiculosus* fucoidan (500 mg/kg per 2 days for 40 days) significantly reduced the hepatic granuloma size and fibrosis response [106]. Lower levels of pro-inflammatory cytokines were observed in the livers of fucoidan-treated infected mice. Infiltration of Treg cells and levels of IL-10 and TGF-β were significantly enhanced in both the livers and spleens from fucoidan-treated mice. Another study aimed to explore the effects of fucoidan from *Fucus vesiculosus* on concanavalin A (ConA)-induced acute liver injury in mice. Pretreatment with fucoidan (10–50 mg/kg for 2 weeks) protected liver function indicated by ALT, AST and histopathological changes by suppressing inflammatory cytokines, TNF-α and IFN-γ [107]. The results demonstrated that fucoidan alleviated ConA-induced acute liver injury via the inhibition of intrinsic and extrinsic apoptosis mediated by the TRADD/TRAF2 and JAK2/STAT1 pathways which were activated by TNF-α and IFN-γ.

Fucoidan from *Fucus vesiculosis* (300–600 mg/kg) was shown to be able to delay the onset and incidence of autoimmune diabetes in non-obese diabetic mice via regulating DC/Treg-induced immune tolerance via induction of IL-10 and TGF-β, while reducing the levels of IL-6 and IFN-γ [108]. In the pancreas TLR4 expression and the downstream molecules were downregulated while pancreatic internal environment was maintained in the fucoidan-treated groups.

In a clinical trial in patients with chronic hepatitis B infection, oral ingestion of a commercial oligo-fucoidan preparation (550 mg twice a day for 48 weeks) was shown to have hepatoprotective effects related to serum concentrations of vitamin D, which is known to have immunoregulatory activity [109]. A clinical trial in healthy volunteers showed anti-inflammatory effects of a blend containing fucoidan from 3 different seaweeds. Daily oral ingestion of 1000 mg for 4 weeks was found to decrease serum IL-6 levels [110]. In advanced cancer patients a mixture of enzymatically digested and undigested fucoidan from *Cladosiphon* *novae* *caledoniae* (4 weeks of 4000 mg/day) was found to reduce several major proinflammatory cytokines, including IL-1β, IL-6 and TNF-α [111]. The analyses revealed that the responsiveness of IL-1β was inversely correlated with overall survival and was suggested as a possible prognostic factor for disease outcome in advanced cancer patients receiving fucoidan.

Taken together, brown seaweed polysaccharide ingestion is shown to be effective in antagonizing the effects of acute and chronic inflammation in both mouse models and clinical trials.

### 4.3. Fucoidan Ingestion and Atherosclerosis in Animal Models

Several studies have reported beneficial effects of fucoidan on outcome of atherosclerosis, a disease related to long-term inflammation of the arterial vessel wall.

ApoE-deficient mice are the most frequently used model for assessing atherosclerotic plaque development. In one study, sulphated polysaccharides from *Laminaria japonica* supplementation markedly reduced the thickness of the lipid-rich plaque, lipid peroxidation and foamy macrophage accumulation in the aorta via suppression of MAPKs and NF-κB signaling [112]. In line, Wang et al. found that *Laminaria japonica* fucoidan (50–100 mg/kg/day for 16 weeks) attenuated atherosclerosis by reducing inflammation and oxidative stress [113]. Furthermore, LMW fucoidan extracted from *Laminaria japonica* inhibited the formation of atherosclerotic plaques; and ameliorated the occurrence and development of atherosclerosis [114]. It decreased the production of inflammatory cytokines and prevented macrophages from developing into foam cells and diminished smooth muscle cells from migrating into the intimal layer of the aorta.

Furthermore, also in other models of atherosclerosis, fucoidan appears to have beneficial effects. In a rat allogenic aorta transplantation model ad libitum ingestion of fucoidan from *Fucus* *vesiculosus* mediated anti-atherosclerotic activity by inhibiting inflammation, suppressing ROS production and down-regulating LOX-1 expression in the vascular wall [115]. In a rat aorta transplantation model fucoidan (LMW) from *Laminaria japonica* (200 mg/kg/day for 35 days) decreased the number of macrophages in the vascular wall by blocking P-selectin activity thereby preventing the development of aortic aneurysms [116]. In the LDLR^−/−^ mouse model of atherosclerosis *Laminaria japonica* fucoidan (50–100 mg/kg/day for 16 weeks) was shown to result in atherosclerosis attenuation by reducing inflammation and oxidative stress [113]. In conclusion, fucoidan appears to be promising in the battle against atherosclerosis by decreasing macrophage infiltration in the vascular wall, as well as by reducing inflammation and oxidative stress.

## 5. Phenolic Compounds and Phytosterols

### 5.1. Phytosterols

Phytosterols, including both sterols, stanols and oxysterols, such as fucosterol, saringosterol and 24-hydroperoxy-24-vinyl-cholesterol, are a group of functional lipid compounds. Compared to other bioactive molecules produced by brown algae, phytosterols exhibit various health-improving effects, especially neuroprotective and anti-inflammatory. Table A3 presents an overview of the significant inflammation-related outcomes in animal models after oral administration of phytosterols. Fucosterol, the most abundant sterol in brown seaweed, when administered in different animal models was found to induce a significant therapeutic effect on injury- or infection-related inflammation. Mo et al. [117] showed anti-inflammatory effects of fucosterol-pretreatment in Concanavalin A-treated mice as a model for acute liver injury. After Concanavalin A-treatment, NF-κB p65 increased markedly and the expression of a nuclear receptor in its upstream pathway, PPARγ, decreased. Both NF-κB p65 and PPARγ are closely related to the release of inflammatory factors such as TNF-α, IL-6 and IL-1β. Fucosterol pretreatment down-regulated the inflammatory response and subsequently necrosis and apoptosis by inhibiting the NF-κB pathway and activating PPARγ.

Anti-inflammatory effects of fucosterol were demonstrated using regular *Sargassum fusiforme* extracts (NH) and enzyme-modified *Sargassum fusiforme* extracts (EH) [22]. Enzyme modification significantly increased the fucosterol concentration in the extract (EH) leading to better results in decreasing pro-inflammatory cytokines as compared to the NH pretreatment group. In addition, both NH and EH reduced the production of NO without inducing any cytotoxicity and even increased cell viability in cultured RAW264.7 macrophages at a concentration of 10 μg/mL or higher. Anti-inflammatory effects of fucosterol were also observed in DNCB-induced NC/Nga mice as a model for atopic dermatitis. Oral administration of fucosterol significantly reduced [22] the scratching behavior of the mice and suppressed the production of pro-inflammatory cytokines (TNF-α and IL-4), resulting in reduced circulating IgE levels.

Bogie et al. showed that 24(S)-Saringosterol, an oxyphytosterol present in *Sargassum fusiforme*, has anti-inflammatory effects likely via activation of liver X receptor (LXR)β in a mouse model of Alzheimer’s disease (AD) [118]. *Sargassum fusiforme* extract rich in 24(S)-Saringosterol activated LXRβ preferentially and to a lesser extent also LXRα. LXRβ plays a key role in the down-regulation of the expression of multiple inflammatory genes [119,120]. AD is characterized by an accumulation of extracellular amyloid-β (Aβ), intracellular neurofibrillary tangles, loss of synapses, neuroinflammation and by a gradual progression of memory loss. After 45 days of dietary supplementation with *Sargassum fusiforme* the formation of Aβ plaques which is related to cognitive decline, was found to be dramatically reduced (~80% reduction) in AD mice. The expression of the LXR-target gene APOE in the central nervous system was increased due to administration of *Sargassum fusiforme* lipid extract. Apolipoprotein (Apo)E increased the clearance of Aβ by microglial cells and suppressed the secretion of Aβ by neurons in vitro. Therefore, the anti-inflammatory effects of 24(S)-Saringosterol may be explained by activation of the LXR-ApoE axis [121]. Similar to 24(S)-Saringosterol-mediated LXRβ activation, fucosterol can activate both LXRα and LXRβ, regulating different aspects of inflammatory gene expression.

### 5.2. Polyphenols

Polyphenols are another class of bioactive compounds from brown seaweed that have attracted great interest in recent years due to their pharmaceutical and biomedical properties. Polyphenols are classified based on their structure. Phlorotannins, highly abundant in brown seaweeds, are polymerized phenolic compounds consisting of phloroglucinol monomer units. Phlorotannins identified in brown seaweed, include eckol, dieckol, phlorofucofuroeckol A. Numerous studies have demonstrated the potential of polyphenol classes as antioxidant, anti-inflammatory, antidiabetic, antitumor, antihypertensive, anti-allergic, hepato-protective and anti-cancer. Table A4 provides an overview of the reported inflammation-related outcomes after oral administration of polyphenols in different animal models and in clinical trials. (Phase 0, 1, 2 and 3).

#### 5.2.1. Polyphenols: Effects in Steady State

The mechanisms underlying the anti-inflammatory effects of polyphenols are complex and are related to various stages of the inflammatory response that are sequential but overlapping. Disturbance of the steady state causes parenchymal tissue and immune cells to respond to injury or irritation through an innate cascade driving inflammation. Irfan et al. [122] demonstrated that phlorotannins strongly inhibit in vivo platelet aggregation in Sprague Dawley rats. In line with this in vitro, phlorotannins downregulated adenosine diphosphate-induced platelet activation (Ca-mobilization, fibrinogen binding, granule release—mediated via decreased Src, PI3K, PLCγ2, MAPK signaling). A clinical trial with 80 overweight participants showed that phlorotannins modestly decrease DNA damage [7], although no significant difference was found in acute phase proteins, anti-oxidant status or in inflammatory cytokines.

#### 5.2.2. Polyphenols: Effects on Allergy

NF-κB is one of the transcription factors that regulates eosinophilic inflammation and IgE-mediated hyperreactivity following allergic inflammation. Oral administration of polyphenols suppressed NF-κB pathway activation, and also inhibited inhibitor kappa B (IκB) that binds to NF-κB [123,124]. Polyphenols were found to alleviate particulate matter-induced airway inflammation in an allergic asthma mouse model [124]. Polyphenol treatment was found to decrease the inflammatory cell count in blood, including eosinophils, neutrophils, basophils. The level of epithelial cytokines, including IL-25, IL-33 and IL-8 also were reduced in the polyphenol-treated mice [124]. Han et al. [123] reported that pretreatment of BALB/c mice, as a model for passive cutaneous anaphylaxis, with Eckol inhibited the production of IL-4, IL-5, IL-6 and IL-13. Moreover, Eckol-treatment suppressed levels of β-hexosaminidase, secreted during the degranulation of mast cells. In addition, oral polyphenol administration was found to reduce the levels of FcεRI on the surface of IgE/bovine serum albumin (BSA)-stimulated mouse bone marrow-derived cultured mast cells (BMCMC). Cross-linking of FcεRI and allergen-specific IgE triggers allergic reactions that may be prevented by polyphenols. These results suggested that Eckol has anti-allergic potential. In a mouse ear-edema model both oral and local administration of phlorotannin, 1-21h prior to irritant application, strongly inhibited arachidonic acid (AA), 12-O-tetradecanoylphorbol-13-acetate(TPA)and immune-mediated (oxazolone (OXA))-induced ear swelling (30–80%) [125]. This suggests that the inhibitory effects of polyphenols are comparable to those of known anti-allergic agents. It was presumed that polyphenols play an anti-inflammatory effect by inhibiting mast cell degranulation, COX-2- and LOX-, and to lesser extent PLA2 activities.

#### 5.2.3. Polyphenols: Effects in Acute and Chronic Inflammation

Inflammation is a beneficial host response for foreign invaders and necrotic tissue with phase 1 being the first, immediate response, typified by ROS production and release of early mediators such as IL-1, TNF-α and arachidonic acid pro-inflammatory metabolites. Once detected extracellularly, ingested microbes will lead to upregulation of TLRs a family of proteins involved in the initial phase of host defense against invading pathogens. TLR4 is the most common member in inflammation phases. Excessive TLR activation, however, disrupts the immune homeostasis by sustained production of pro-inflammatory cytokines and chemokines.

Polyphenols were demonstrated to be very well capable of suppressing the increase of TLRs, including TLR4 [124,126,127], TLR2 [124,127] and TLR7 [124]. TLRs as primary sensors of microbial products activate signaling pathways that lead to the induction of immune- and inflammatory genes, such as the NF-κB pathway. Polyphenol treatment also significantly decreased NF-κB and thereby the modulation of inflammation-related signaling cascades [123,124,126,127,128,129,130,131].

ROS are a crucial factor in the inflammatory response, playing multiple roles after tissue injury, including initiation of acute inflammation, clarifying infection and necrotic tissue and mediation of various intracellular signal transduction pathways. Anti-inflammatory effects of polyphenols via antioxidant activities were demonstrated by Kang et al. [132]. They found that serum ferric reducing antioxidant power (FRAP) significantly increased 30 min after polyphenol treatment in the Sprague Dawley rat-model but declined quickly thereafter. Polyphenols showed anti-inflammatory effects by reducing the expression of ROS [131,133,134,135]. Administration of the polyphenol-rich fraction of *Ecklonia cava* reduced ROS and NO generation in LPS-stimulated inflammation in zebrafish [133]. ROS can activate a variety of transcription factors leading to the differential expression of genes involved in inflammatory pathways. On the other hand, excessive production of ROS can cause irreversible damage to DNA. Due to this dual effect, polyphenols often show a crucial effect in tumor models by upregulating ROS in tumor cells and at the same time downregulating ROS in healthy cells. Yang et al. [129] indicated that oral administration of phlorotannins to the SKOV3-bearing mouse model of ovarian cancer enhances cancer cell apoptosis via upregulation of the ROS pathway but protects against healthy kidney cell damage by downregulating ROS levels. Tissue damage leads to a rapid increase in ROS which stimulates PGE2 production via the activation of COX-2. Polyphenols exert anti-inflammatory effects not only by suppressing COX-2 [128,130,131,136], but also by reducing PGE2 production [130,131] which exacerbates inflammatory responses and immune diseases. The production of early inflammatory cytokines such as TNF-α, IL-6 and IL-1β, that is increased in phase 1, was significantly inhibited by oral administration of polyphenols in vivo, in different mouse, rat and zebrafish models [124,126,127,128,130,131,135,137,138,139], and also in vitro, in RAW264.7 macrophages [131,134]. Polyphenol treatment [128,138] decreased the expression of CCL2/MCP-1 and consequently may reduce the infiltration of macrophages and subsequently inflammation [126,128].

In phase 2, the regulation of inflammation is amplified via positive feedback and adaptive immunity is activated. Oral administration of polyphenols significantly affects macrophage infiltration and the balance of macrophages with an M1 or M2 phenotype. Oral administration of polyphenols decreased the expression of CD11b and CD80, markers for M1 macrophages [126,127,137,138]. The M2 type is identified by marker CD206, and can prominently expresses IL-10, a cytokine with potential anti-inflammatory effects and plays an important role in limiting the host immune response to pathogens. Polyphenol treatment was found to increase the expression of the M2 markers, CD206 [126,127,137,138] and IL-10 in acute liver injured mouse model and HFD with or without seaweed supplement mouse model [124,127,135,138]. Oral administration of polyphenols induced a decrease in the level of iNOS [130,131,134,138] and the levels of NO in both.

#### 5.2.4. Polyphenols: Late Consequences of Inflammation and Sequels

Oral administration of polyphenols may be promising for the treatment of severe or chronic inflammation and its consequences (phase 3). Oral administration of polyphenols resulted in a reduction in food intake and in body weight [126,127,128,137,140], as well as in the storage of triglyceride (TG) and total cholesterol (TC) [128,137]. After oral administration of extracts rich in polyphenols, also the leptin/adiponectin ratio, an important marker for inflammation and obesity, decreased [128]. Choi et al. and Son et al. [127,137] showed that the production of receptor for advanced glycation end-products (RAGE), closely related to inflammation and visceral fat hypertrophy, and RAGE-RAGE ligand binding was reduced in obese individuals treated with polyphenols. In obesity-associated type 2 diabetes, low-grade chronic inflammation can lead to an increase of blood glucose levels and to insulin resistance. Polyphenol treatment improved insulin sensitivity [128] and suppressed the increase of blood glucose levels in high-fat diet-induced obese mice [140]. A novel derivative from phloroglucinol called Compound 21 significantly exerted protective effects on multiple sclerosis through promotion of remyelination and suppressing neuroinflammation in a cuprizone-induced mouse model for multiple sclerosis [141]. In another study these authors showed that Compound 21 reduced the population of Th1/Th17 cells and inhibited their infiltration into the CNS. These results indicated a potential neuroprotective effect of Compound 21 [142].

In conclusion, polyphenols have various therapeutic effects including anti-inflammatory, anti-obesity, anti-diabetic and antioxidant. Polyphenols may be a highly promising treatment strategy for diseases involving chronic low-grade inflammation, but further clinical studies are needed.

## 6. Fucoxanthin(ol) and Meroterpenoids

Fucoxanthin is a major marine carotenoid present in chloroplasts of brown seaweeds, and particularly seaweeds such as *Undaria pinnatifida*, *Laminaria japonica* and *Sargassum honeri* are rich in fucoxanthin [143]. Ingested fucoxanthin is metabolized to fucoxanthinol in the small intestine and then absorbed [144]. Therefore, fucoxanthinol has a higher bioavailability than fucoxanthin. Multiple potentially beneficial health effects have been ascribed to both fucoxanthin and fucoxanthinol; e.g., anti-oxidative, anti-inflammatory, anti-obesity, anti-diabetic and anti-carcinogenic properties [145,146]. Meroterpenoids are partially derived from a terpenoid pathway (mero- means partial). Tetraterpenoids, of which carotenoids are the most common representatives, belong to the terpenoids and consist of eight isoprene units [147]. Meroterpenes can be isolated from brown algae such as *Sargassum serratifolium*. The meroterpenoid-rich fraction from the ethanolic extract (MES) of this brown alga is known for its antioxidant and anti-inflammatory activities [148]. Table A5 presents an overview of the significant outcomes of studies investigating oral administration of fucoxanthin, fucoxanthinol or MES in animal models related to the inflammatory response.

### 6.1. Fucoxanthin(ol) and Meroterpenoids: Effects in Acute and Chronic Inflammation

Fucoxanthin is known for its antioxidant potential through its ability to scavenge radicals effectively and to enhance enzymatic antioxidant activity [149]. Enhanced activity of antioxidant enzymes superoxide dismutase (SOD), catalase (Cat) and glutathione peroxidase (GPx) was observed in plasma and testis of rats [150], mice [151] and hamsters [152] after treatment with fucoxanthin. Fucoxanthin reduced the increased production of ROS as a consequence of increased oxidative stress and reduced the increased malondialdehyde formation during lipid peroxidation, which in turn causes upregulation of pro-inflammatory cytokine production [153,154]. Malondialdehyde levels were reduced in plasma, sperm and/or testicular tissue of rats [150], mice [151,155] and hamsters [152] after fucoxanthin treatment. Additionally, a reduction of ROS, such as superoxide (O_2_^−^) and hydrogen peroxide (H_2_O_2_) was seen both in vivo and in vitro after oral administration of fucoxanthin [150,151,152,155].

Oral administration of fucoxanthin in different animal models decreased the expression of various pro-inflammatory mediators, including cytokines such as TNF-α, IL-6 and IL-1β. This was observed in white adipose tissue, plasma, testis and colonic tissue after stimulation with various inflammatory triggers [150,151,155,156]. Sugiura et al. demonstrated the anti-inflammatory and inhibitory effects of oral or percutaneous administration of fucoxanthin on mouse ear swelling induced by different irritants [157]. Fucoxanthin and fucoxanthinol were shown to inhibit the enzymatic activities of PLA_2_ and COX-2, thus restraining the generation of pro-inflammatory arachidonic acid metabolites in these mice. These anti-inflammatory effects of fucoxanthin and fucoxanthinol were also confirmed in vitro using rat basophilic leukemia cells, which showed reduced mRNA expression of sPLA_2_ and COX-2 upon treatment with both fucoxanthin and fucoxanthinol [157]. In addition, Tan et al. found that COX-2 and iNOS mRNA expression were downregulated in obese mice upon fucoxanthin administration [155]. Similarly, Maeda et al. demonstrated decreased expression of MCP-1 in white adipose tissue in mice with obesity-related inflammation upon treatment [145,156]. Since MCP-1 is a pro-inflammatory cytokine this suggests an anti-inflammatory effect of fucoxanthin on adipocytes. In mouse models of DSS-induced colitis and colitis-associated colon carcinoma, a reduction in total NO content and in NO release in colonic tissue was observed after oral administration of fucoxanthin [151]. Moreover, NO production was also reduced after treatment with fucoxanthin in cisplatin-induced testicular damage in hamsters [152]. Additionally, the meroterpenoid-rich fraction of an ethanolic extract from *Sargassum serratifolium* (MES) induced anti-inflammatory activities in high-cholesterol-fed mice. The mice demonstrated decreased serum levels of MCP-1 and keratinocyte chemoattractant, which are pro-inflammatory chemokines causing monocyte adhesion in vascular lesions. Furthermore, MES supplementation resulted in reduction of ICAM-1, VCAM-1, MCP-1, COX-2 and MMP-9 expression in aortic tissue. These results indicated that MES prevented vascular inflammation in these mice [148]. Similarly, mice fed a high-fat diet supplemented with MES, compared to un-supplemented high-fat diet showed decreased expression of macrophage markers F4/80 and MCP-1, indicating a suppression of inflammation [158].

### 6.2. Fucoxanthin(ol) and Meroterpenoids: Late Consequences of Inflammation and Sequels

Related to the inflammation in adipose and other tissues, oral fucoxanthin supplementation was also shown to have effects counteracting obesity and obesity-related morbidity [159]. Administration of fucoxanthin to mice fed a high-fat diet reduced gain in body weight and in weight of white adipose tissue as compared to chow-fed control mice [155,156]. Furthermore, Maeda et al. demonstrated that mice fed a high-fat diet also receiving fucoxanthin displayed significantly lower plasma levels of LDL-cholesterol and leptin compared to mice that were fed a high-fat diet only, indicative of moderated metabolic dysregulation [156]. Additionally, the obesity-related reduction in expression of beta-3 adrenergic receptor (ADRB3), responsible for lipolysis and thermogenesis [160], appeared significantly restored in mice upon addition of fucoxanthin to their high fat diet [156]. Moreover, Tan et al. showed a decrease in myeloperoxidase (MPO) activity in mice with high-fat diet-induced obesity after oral fucoxanthin administration, which implies a reduction in polymorphonuclear cell infiltration [155]. In addition, MES supplementation suppressed body weight, TG, glucose and free fatty acid concentrations in plasma of high fat diet-fed mice. In addition, the lower HDL cholesterol levels increased to comparable levels as in the control group. Moreover, increased expression of UCP-1 and ADRB3 in subcutaneous tissues demonstrates that MES supplementation causes conversion of white to beige/brite adipocytes which resembles brown adipose tissue [158]. These results suggest anti-obesity effects and inhibition of lipogenesis by MES supplementation.

In line with improvement of metabolic functions induced by fucoxanthin supplementation, anti-diabetic effects of fucoxanthin have been observed [145,150,156]. Feeding a high fat-diet containing fucoxanthin resulted in decreased plasma insulin and blood glucose levels in mice, to levels similar as in mice fed a regular diet [156]. Moreover, mRNA levels of GLUT4, encoding the insulin-sensitive glucose transporter in adipose tissue and muscle, were restored to normal levels when the high fat diet was supplemented with fucoxanthin [156]. In the diabetic KK-Ay mouse model fucoxanthin consumption was found to decrease elevated plasma blood glucose concentrations [145]. It was also shown that glucose intolerance improved by fucoxanthin dietary addition [145]. More recently, Kong et al. found that treatment of diabetic rats with fucoxanthin significantly reduced levels of plasma glucose compared to diabetic rats without any treatment [150]. Insulin concentrations and homeostatic model assessment of insulin resistance (HOMA-IR) levels were significantly reduced in these rats. Finally, fucoxanthin supplementation inhibited the expression of the suppressor of cytokine singaling-3 (SOCS-3), involved in the induction of insulin resistance [150]. Together, these results indicate that fucoxanthin possesses anti-diabetic effects by suppressing inflammation and thereby improving insulin sensitivity.

## 7. Concluding Remarks

In this comprehensive systematic review, we aimed to provide an overview on the modulating role of intake of complete brown seaweed, its extracts or selected compounds on the modulation on different aspects of the inflammatory immune response. This includes the impacts of seaweed consumption on steady state immune parameters, effects on allergies, the innate and adaptive immune response and also on chronic, low-grade inflammation. Brown seaweeds constitute a group of approximately 2000 species containing several common but also unique bioactive molecules with immunomodulatory functions. We therefore distinguished the impact of four different categories of compounds, i.e., polysaccharides, (poly)phenols, phytosterols and carotenoids.

**Figure 2:**
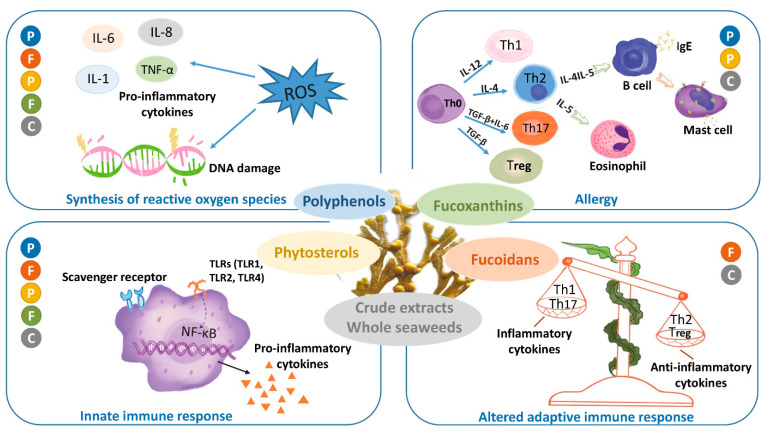
Overview of the general effects of crude brown seaweed or its extracts (C, grey) and of its constituents: polyphenol (P, blue), Fucoxanthin (F, green), Fucoidan (F, brown) and Phytosterols (P, yellow) on the different phases of the inflammatory processes, including allergy. The colored dots showing the crude seaweed or its extract and its constituents in the separate boxes indicate in which phases of the immune response they exert their effects.

We identified three common denominators across the effects of brown seaweed and constituents thereof on inflammation (represented in Figure 2). Firstly, most purified compounds, despite their diverse chemical nature, appear to inhibit similar aspects of inflammation, in particular synthesis of reactive oxygen species. This common effect is rather puzzling. Yet, a caveat of some studies may be the use of compound concentrations beyond physiological levels. In addition, purification of the compounds used in the cited studies mostly left sufficient room for concentrations of contaminants that might contribute to, or even explain, the claimed effects. In our view, this calls for rigorous comparative research of the various compounds in an identical experimental setting. Nevertheless, the suppression of reactive oxygen species provides an interesting angle to study immune-modulatory effects of brown seaweed constituents.

Secondly, brown seaweeds interfere with the innate immune response on the level of TLR-induced NF-κB signaling. This route is actually linked with the previous one since oxygen radicals drive and amplify innate NF-κB-mediated activation. In conjunction, a plethora of in vitro and in vivo experiments support a suppression of IL-6, IL-1, iNOS and TNF-α upon treatment with brown seaweeds. Polyphenols, fucosterol, fucoxanthin and fucoidan all seem to be active in this. Yet, when fucoidan was applied in the pretreatment setting, it was also shown to potentiate the NF-κB axis to reduce susceptibility to infection via scavenger receptor A and TLR4 activation in an antibiotic-like fashion. These seemingly contradictory findings underscore the versatile properties of brown seaweed constituents in the modulation of the innate immune response. Therefore, interpretation should be performed with care when extrapolating in vitro findings to human applications. Nevertheless, most in vivo studies summarized in this review show unequivocal anti-inflammatory effects. The mechanistic background of brown seaweed health benefits probably goes beyond direct inhibition of inflammation. Multiple studies indicate that brown seaweed compounds interact with pathways and processes involved with energy sensing and survival, such as AKT/mTOR/AMPK and autophagy. Thus, brown seaweed compounds might also function as caloric restriction mimetics and thereby stimulate vitality.

Thirdly, the adaptive immune response displays an altered Th1/Th2 response in response to brown seaweed. Different constituents have been identified to skew the Th1/Th2 balance. Depending on the molecular weight of the fucoidan, different outcomes have been identified. Fucosterol has been shown to skew Th0 cells into Th2 cells in a model for allergy, whereas polyphenols suppress the Th1/Th17 response in an animal model for MS. The net effect of brown seaweed on Th1/Th2 skewing cannot be generalized and is largely dependent on the composition of the different constituents in the seaweed. On the level of allergy, Th2 suppression reduces IL-4 cytokine levels, decreased IgE production and suppressed mast cell activity.

Inflammation is a universal response of the body to damage, infection or otherwise disturbed homeostasis. For this review, we have restricted the search terms to those that are related to inflammation, allergy and immunity. The broad implication of the inflammatory response in the maintenance of bodily integrity, however, entails that several studies that focus on aspects only indirectly related to inflammation, were not included in the final result, whereas similar studies were, based on different choices by the respective authors for their specific wording. Nevertheless, we are confident that this review covers the main aspects of oral supplementation of brown seaweeds and their components on aspects of inflammation, allergy and immunity in a broad sense. More well-designed human studies applying individual seaweed constituents as well as whole seaweed (extracts) will provide more insight into the applicability of brown seaweed as immune-modulatory nutritional intervention strategies.

## Figures and Tables

**Table 1:** Phases of the inflammatory response with characteristic features ^(1)^.

Phase	Description	Characteristic Read-Outs
0 steady state	homeostatic condition	- growth, organ weight, etc. - leukocyte numbers and subset composition - steady state activities, e.g., phagocytosis
1 short-term	initiation of inflammation as response to triggering by damage- or infection-related molecules	- mast cell and basophil degranulation - neuronal activation (e.g., scratching) - reactive oxygen species (ROS) - pro-inflammatory arachidonic acid (AA) metabolites (e.g., PGE_2_) - phospholipase (PLA_2_), cyclo-oxygenase (COX-2/PTGS2) and lipoxygenase (LOX/ALOX) activity - early pro-inflammatory cytokines (IL-1, TNF, IL-6 ^(2)^)
2 short-term	amplification and regulation of inflammation; initiation of adaptive immunity	- additional pro- and anti-inflammatory cytokines (e.g., IL-12, IL-10, IL-2) - soluble forms of cellular R (e.g., sCD25, sCD163) - chemokines (e.g., CXCL8, CCL2) - induction of iNOS and nitric oxide (NO) production - anti-inflammatory AA metabolites (e.g., LXA4) - endothelial activation - edema - leukocyte mobilization and tissue infiltration - acute phase proteins (e.g., CRP) - HPA-axis activation (cortisol or corticosterone) - lymphocyte proliferation - microbial infection parameters - clearance and repair in short-term
3 short- or long-term	consequences of severe or chronic inflammation	- clearance and repair in long-term - glucose, insulin resistance, diabetes - adipokines, leptin resistance, obesity - glucocorticoid resistance, stress, hampered down-regulation of inflammation - inflammation-induced malignancy

^(1)^ See Box 1 for a brief introduction on inflammation. ^(2)^ In the inflammatory response, IL-6 is produced in a second wave, as it has to be expressed de novo, while initial TNF and IL-1β release only requires processing (i.e., membrane cut or enzymatic cleavage and secretion, resp.). Yet, IL-6 is frequently measured together with IL-1β and TNF. Therefore, IL-6 is classified in the first phase. In itself, it is an inducer of mediators of the second phase, in particular acute phase proteins.

## Supplementary Materials

The following are available online at https://www.mdpi.com/article/10.3390/nu13082613/s1, Supplementary Figure S1: Flowchart of systematic search and search strategies.

## Appendix A

**Table A1:** Effects of oral supplementation of whole brown seaweeds or crude extracts on inflammation-related parameters.

Compound/Format	Effective Concentrations	Algal Source	Experimental Model	Inflammatory Phase ^1^	Significant Findings ^2^	Reference
EtOH extract	300–2000–5000 mg/kg; single dose	*Sargassum micracanthum*	BALB/c mouse; toxicity; follow-up 2 weeks	steady state	non-toxic up to 5000 mg/kg	[3]
EtOH-H_2_O extracts	single dose LD50 = 500–2000 mg/kg; oxidative stress reduction: 200 mg/kg/day, 4 weeks	*Fucus vesiculosus*	Swiss mouse; Sprague Dawley rat; toxicity: acute anti-oxidant activity: 4 weeks	steady state	LD50 acute toxic > 750 mg/kg 4 week supplementation: ↓ 13% food intake in 4 week 30–50% ↓ WBC but no change in differential counts 20–25% ↑ in liver and kidney weight ↑ PON-1 activity (protects against ox-LDL) ↑ SOD	[5]
MeOH-, hexane- or chloroform-extract	>2000 mg/kg, single dose	*Cystoseira compressa (Esper)*	male and female albino mouse; toxicity	steady state	no lethality in oral testing (2000 mg/kg)	[4]
EtOH extract, polyphenol-rich ultrafiltrate	100 mg/day, 8 weeks	*Ascophyllum nodosum*	human, BMI ≥ 25	steady state	marginal ↓ lymphocyte DNA damage in only obese =CRP =cholesterol, HDL, LDL, TG anti-oxidant =in vitro LPS-/TPA-induced pro-inflammatory cytokines in monocytes, lymphocytes =total plasma peroxide	[7]
whole, dried	4.8 g/dayay, 4 weeks	*Sargassum muticum*	human	steady state	↓ oxLDL (14%); correlated to ↑ total antioxidant status ↑ NK count; =lymphocyte count ↓ fatigue ↑ liver function (↓ AST, ALT)	[6]
commercial extract	2% in feed, 3 weeks	*Ascophyllum nodosum*	Spanish and Boer × Spanish goat; stress by transport and feed withholding	steady state	=cortisol =WBC and subset counts, but ↓ Eosinophil =phagocytosis ↓ lipid peroxidation; ↓ SOD (strain diff.) ↑ glutathione peroxidase (10–15%)	[8]
commercial extract	0.5% in feed; 41 weeks	*Ascophyllum nodosum*	Lohmann LSL-Lite and Lohmann Brown-Lite hens, heat stress	steady state	short-term↓ feed intake; ↑ feed/egg efficiency strain-dependent ↑ production, ↑ feed efficiency, ↑ heat stress resistance, improved ALP, ALT, GGT liver parameters	[161]
seaweed powder + anti-bacterial peptides (cecropin)	1–5% of basal diet	*Laminaria japonica*	Arbor Acres broiler chicks	steady state	synergistic effect of seaweed + cecropin: ↑ antibodies, ↑ lymphocytes, microbiota: ↑ *Lactobacillus*, ↓ *E.coli* ↑ feed conversion ratio	[9]
hot water extract	100–500 mg/kg in feed, 12 weeks	*Sargassum oligocystum*	fish, Pangasius *(Pangasianodon hypophthalmus)*	steady state	↑ weight, daily growth rate, feed conversion ratio ↑ WBC, RBC, Hb, Hc, platelets	[10]
seaweed meal	3–6–9% in feed, 6 weeks	*Sargassum ilicifolium*	fish, Asian sea bass *(Lates calcarifer)*	steady state	↑ growth; ↑ pancreatic enzyme activities ↑ serum Ig; (alternative) complement pathway components, lysozyme ↑ Ig in skin mucus ↑ liver SOD, IL-1β mRNA	[11]
whole seaweed	10% in feed, 8 weeks	*Sargassum ilicifolium*	fish, great sturgeon *(Huso huso)*	steady state	↑ growth ↑ serum protein, lysozyme, IgM, respiratory burst, complement ↑ Hb, RBC, WBC =TG, LDH, AST, ALT ↓ blood cholesterol ↑ survival upon *Yersinia ruckeri* infection (14 days infection)	[12]
aqueous extract	400 mg/kg, 8 weeks	*Sargassum angustifolium*	fish, rainbow trout *(Oncohrynchus mykiss)*	steady state	↑ weight gain ↑ Hb, Hc, RBC, WBC, total protein, albumin↑ survival and immune response to *Yersinia ruckeri* infection	[13]
EtOH extract	200 mg/kg, 31 days	*Sargassum horneri*	male C57BL/6 mouse; in vivo anti-DNP-IgE i.v. + DNP i.n. challenge	allergy	↓ nasal rubbing ↓ mast cell degranulation Proposed mechanism: Chlorophyll-C2 →↓ PI-3K + ↓ Btk, ↓ Syk active upon FcεR trigger →↓ Ca-mobilization	[15]
EtOH extract	10, 50, 100 mg/kg, 1×/day, 3 weeks	*Sargassum horneri*	female NC/Nga mouse; house dust mite/dayNCB skin sensibilization DNCB skin challenge (2×/week)	allergy	↓ atopic dermatitis symptoms ↓ epidermal hyperplasia, hyperkeratosis, skin dryness ↓ mast cell + eosinophil skin infiltrates ↓ IL-25, IL-33 → ↓ Rantes (CCL5), Eotaxin (CCL11), TARC (CCL17) in skin ↓ IL-4, -5, -13, ↓ IL-6, -10, IFN-γ in serum ↓ spleen size increase ↓ IgG1, IgG2a increase	[16]
EtOH extract	100–300 mg/kg/day in diet, 5 weeks	*Costaria costata*	male NC/Nga mouse; DNCB-induced atopic dermatitis skin allergy	allergy	↓ inflammatory cell and mast cell infiltration, epidermal thickness, erythema, hemorrhage, dead skin cell layers, skin dehydration ↓ Eotaxin and TARC (CCL17)↓ serum IgE and histamine normalization of spleen lymphocyte proliferation and cytokine production	[17]
water extract	100–300 mg/kg/day, 4 weeks	*Laminaria japonica*	female NC/Nga mouse; DNCB-sensitized and challenged dorsal skin	allergy	↓ dermatitis severity; ↓ inflammatory mediators ↑ skin moisture in vitro: ↓ p38 MAPK,↑ ERK, ↓ STAT1 in HaCaT human keratinocyte cell line	[18]
dried powder	1–5–10%, 6 weeks	*Eisenia* *(=Ecklonia) arborea*	female Brown Norway rat; oral OVA immunization	allergy	↓ OVA-specific IgE, ↓ total IgE (n.s. with 10% diet) ↓ serum histamine ↑ IFN-γ in spleen and MLN ↓ IL-10 in spleen and MLN	[14]
EtOH extract	0.1–0.3 g/kg/day, 3 days prior to experiment	*Laminaria japonica*	male SD rat; carrageenan-induced paw inflammation	1,2	↓ paw swelling, leukocyte infiltration in vitro: ↓ IκB phosphorylation → ↓ iNOS, COX2, TNF-α, IL-1β, IL-6	[19]
diethylether fraction	0.1–1 mg/mouse, 2×	*Sargassum fusiforme*	ICR mouse; ear-swelling after irritant	1,2	↓ ear swelling by arachidonic acid, TPA, oxazolone in vitro: ↓ mast cell degranulation by inhibiting PLA2, COX2, LOX, HA	[20]
ethyl acetate extract	200 mg/kg, 5 weeks	*Eisenia bicyclis = Ecklonia bicyclis*	male SD rat; isolated peritoneal Mϕ	1,2	↓ LPS-induced iNOS expression and NO production ↓ NF-κB nuclear translocation with and without LPS =(LPS-induced) tumoricidal activity against B16 melanoma	[21]
EtOH extract +/− enzyme-treatment	50–200 mg/kg, 3 weeks	*Hizikia. = Sargassum fusiforme*	male C57BL mouse; LPS-stimulation of isolated peritoneal Mϕ conA-induced splenic lymphocyte proliferation	1,2	↑ LPS-stimulated IL-1β, TNF-α, IL-6 by peritoneal Mϕ ↑ conA-induced splenocyte proliferation	[22]
EtOH extract	6–24 mg/kg/day, 4 days	*Sargassum serratifolium*	male ICR mouse; LPS i.p. (2 mg/kg), blood sampling after 2 h	1	↓ TNF, IL-1β, IL-6 confirming in vitro findings	[23]
enzymatic extract	50–200 μg/mL, 1 h prior to and during exp	*Sargassum polycystum* *Chnoospora minima*	zebrafish embryos; in vivo 24 h challenge w/H_2_O_2_ (10 μg/mL) or LPS (10 μg/mL), monitored 5d	1,2	↓ H_2_O_2_-induced ROS levels and cell death ↓ LPS-induced ROS, NO and cell death	[24]
whole seaweed	0–4% feed, 40 days	*Sargassum latifolium*	Barki sheep *(Ovis aries)*; i.v. LPS challenge (1.25 μg/kg), after day.28 + day.35	2	↓ body temperature, respiration rate ↓ leukocytosis, ESR, HSP70 ↑ blood anti-oxidant capacity (CAT, SOD) ↓ damage-related molecules: malondialdehyde (MDA; lipid peroxidation product); ALAT, LDH	[25]
whole seaweed	0–4% feed, 40 days	*Sargassum latifolium*	Barki sheep (*Ovis aries)*; heat stress (solar experiment 8–17) vs. mild temperature without solar exposure	2,3	↓ Δ leukocytosis, Δ ESR ↓ proinflammatory cytokines, HSP70 ↑ body weight gain, kidney function, blood anti-oxidant function	[26]
commercial extract	1% feed, 27 days prior to and during exp (10 days)	*Ascophyllum nodosum*	crossbred wether lamb (*Ovis aries*); heat stress	1,2	↓ heat stress-induced reduction of phagocyte oxidative burst ↑ SOD ↓ heat stress-induced changes in GSH-peroxidase activity ↓ lipid peroxidation ↑ leukocyte phagocytosis	[27]
EtOH and organic-purified fraction	15 mg/kg/day, 6 weeks	*Turbinaria ornata*	female C57BL/6J mouse; DSS-induced colitis	1,2	↓ disease activity index ↓ histopathology incl. length reduction, neutrophil infiltrate ↓ TNF ↑ FoxP3, Treg, but = Th17 ↑ IL-10	[28]
aqueous extract (AE) + probiotic mix	100–300 mg/kg, 2×/day for 7 days	*Laminaria japonica*	male BALB/c mouse; DSS-induced colitis	1,2	AE alone: ↓ colitis, incl. ↓ colonic IL-1. IL-6 AE + probiotics: synergistic ↓ colitis, ↓ IL-1, -6, -12p40; not IFN-γ, IL-10, IL-12p70	[29]
EtOH-extract	50–200 mg/kg/day, d.28–98	*Sargassum muticum*	male DBA/1J mouse; collagen-induced arthritis	1,2,3	↓ arthritis and edema ↓ IL-6, TNF, IFN-γ in serum ↓ joint degradation, inflammatory cytokines in joints possibly explained by apo-9′ fucoxanthinone (sim. effects)	[30]
GFS = hot water extract with galactofucan sulfate	therapy: 4 × 560 mg/day, 10 days, 1–24 mo. maintenance: 2 × 560 mg/day	*Undaria pinnatifida*	human; response in patients with active or latent Herpes infection	2	15/15 patients with active disease: ↓ symptoms or full clearance of infection no side effects inhibition of relapse in pts with latent disease	[31]
hot water extract, and HCl-EtOH extract	100 mg/kg/day (heat extract), 10 mg/kg/day (HCl-EtOH extract), for 3–7 days	*Laminaria japonica*	kuruma shrimp (*Marsupenaeus* *japonicas*) in vivo WSSV infection in vitro hemocyte analysis	1,2	↑ survival upon WSSV infectionhemocyte fMet-Leu-Phe stimulation: ↑ chemotaxis ↑ superoxide production ↑ phenol oxidase activity ↑ phagocytosis	[32]
hot water-EtOH extract	20–200 mg/kg; 7 weeks	*Hizikia. = Sargassum fusiforme*	male SD rat; ligature-induced periodontitis	3	↓ alveolar bone loss due to inflammation	[33]
hot water extract of 10 different herbs incl. *S. fusiforme*	10 mg/kg; 10 weeks	*Sargassum fusiforme*	female SD rat; induced autoimmune thyroiditis by CFA-IFA thyroglobulin immunization	3	↓ AI-thyroiditis (cellular infiltrate), ↓ auto antibodies	[34]
whole seaweed, freeze-dried, powdered	5% in chow, 8 weeks	*Undaria pinnatifida*	male C57BL/6J mouse, HFD^+/−^ seaweed supplement	3	↑ total plasma cholesterol (cf. HFD) fecal cholesterol excretion ↓ ↓ MCP-1 induction obese phenotype not prevented no glycemic improvement in i.p. glucose tolerance test microbiome composition (HFD + seaweed) is closer to LFD than to HFD	[38]
whole seaweed, freeze-dried	5% in chow, 16 weeks	*Undaria pinnatifida* (*Up*), *Laminaria japonica* (*Lj*), *Sargassum fulvellum* (*Sf*), *Hizikia =* (*Sargassum*) *fusiforme* (*Hf*)	male C57BL/6N mouse, HFD^+/−^ seaweed supplement	3	body weight or adiposity: no change cf. HFD, except HFD+Up →↑ weight + ↑ subcutaneous adipose tissue adiponectin: ↓ with HFD+Up leptin: ↓ with HFD+Lj, +Sf, +Hf insulin resistance: ↓ w/HFD+Lj blood glucose: ↓ HFD+Lj or +Hf = LFD; HFD+Up = HFD crown-like structures in adipose tissue: ↓ ↓ with all; =LFD LPS-induced pro-inflammatory cytokines by BMDM: ↓ with all	[36]
MeOH extract + carob pod	0.1%/0.9%, 4 weeks	*Undaria pinnatifida*	male Wistar rat, MetS after 8 weeks western diet	2,3	=body weight, fat mass or muscle =food or energy intake ↓ systolic blood pressure at low dose; ↑ at high dose =CRP ↑ insulin, =glucose ↓ non-esterified fatty acids	[37]
hot aqueous extract	500 mg/kg/day started after 2 weeks, for 2 weeks	*Ecklonia cava*	female Sprague Dawley rat, letrozole-induced PCOS	2,3	↓ vaginal leukocyte infiltration restore normal estrous cycle restore normal plasma hormonal levelsnormalize expression of gonadotropin- and steroid hormone-related genes = weight gain upon PCOS induction	[39]

^1^ Phases of inflammation as indicated in Table 1: 1-initiation of inflammation; 2-short term amplification and regulation; 3-short- or long-term consequences. ^2^ Symbols used: ↑—increase; ↓—decrease; =—no change.

## Appendix B

**Table A2:** Effects of oral supplementation of brown seaweed derived polysaccharides on inflammation-related parameters.

Compound/Format	Effective Concentrations	Algal Source	Experimental Model	Inflammatory Phase ^1^	Significant Findings ^2^	Reference
Fucoidan	0.2%, 2% fucoidan chow for 8 weeks	*Cladosiphon okamuranus*	Rats	steady state	Uptake through intestinal tract	[46]
Fudoidan 7 kDa (LMW)	2000 mg/kg, Single dose	*Ascophyllum nodusum*	rats	steady state	No toxicity	[50]
Sodium-Alginate (SA)	5% SA containing chow for 6 weeks	Unknown	Male C57BL/6, Non-alcoholic steatosis model	steady state	↑ Intestinal barrier function in small intestine ↓ Hepatic lipid accumulationLiver: ↓ TNFα, ↓ Collagen-1α1 Liver: ↓ Macrophage infiltration	[162]
Fucoidan-rich extract	50–100–150 mg/kg for 14 days	*Undaria pinnatifada*	In vitro: RAW 264.7 In vivo: C57BL/6 mice	1,2	In vitro: ↑ NO, ↑ TNF-α, IL-1α, IL-1β, IL-6 In vivo: ↑ CD3+, CD4+ ↑ TNF-α, IFN-γ ↑ IgM	[163]
Fucoidan	10–100 mg/kg for 21 days	*Cladosiphon tokida*	In vitro: RAW 264.7 + S180 tumor cells In vivo: S180 tumor bearing mice	2	↑ NO-production by macrophages↑ NK-cell mediated cytotoxicity ↑ NF-κβ translocation in macrophages	[52]
Fucoidan-rich extract	50–100–150 mg/kg for 14 days	*Undaria pinnatifada*	Cyclophosmamide-immunosuppressed male C57BL/6 mice	1,2	All doses: ↑ NK cytotoxicity 100–150 mg/kg: ↑ proliferation of T cells 150 mg/kg: ↑ TNF-α, IgM and total IgG	[93]
Extracted polysaccharides (SFP)	100–200–300 mg/kg, 14 days	*Sargassum fusiforme*	Cyclophosmamide-immunosuppressed ICR mice	1,2	Compared to the cy model group: ↑ Thymus and spleen indices Liver: ↑ SOD, ↓ MDA, ↑ GSH ↑ villus height in normal mice, = in CY-treated mice ↑ intestinal epithelial lymphocyte and Goblet cells in normal and CY-treated mice	[164]
Fucoidan	Buccal LPS injection on 3 separate days followed by fucoidan ingestion *P. gingivalis* infection day 10–25, followed Fucoidan ingestion day 29–49	*Sargassum wightii*	Mouse–Buccal LPS and bacterial (*P. gingivalis*) induced inflammation In vitro: RAW 264.7—LPS induced inflammation	2	↓ TNF-α, IL-1β, IL-6 ↓ monocyte and dendritic cell recruitment =lymphocyte numbers ↓ IL-17, ↑ IL-10 ↓ inhibited antigen-specific immune response No inhibition of bacterial induced periodontitis In vitro: ↓ iNOS, COX-2 ↓ NO, PGE2 ↓ TNF-α, IL-6, IFN-γ	[55]
Sulfated polysaccharide >30 kDa	25–50–100 μg/mL	*Sargassum horneri*	Zebrafish embryos—LPS induced inflammation In vitro: RAW 264.7—LPS induced inflammation	1,2	↓ LPS-induced NO production, toxicity, cell death In vitro: ↓ p-IKβ ↓ iNOS, COX-2 ↓ NO, PGE2 ↓ TNF-α, IL-6	[56]
Fucoidan	25–50–100 μg/mL	*Fucus vesiculosus*	Zebrafish embryos—LPS induced inflammation In vitro: RAW 264.7—LPS induced inflammation	1,2	↓ NO, ROS production ↓ neutrophil and macrophage recruitment dose dependent activity In vitro: ↓ iNOS, COX-2 ↓ NO, PGE2 ↓ TNF-α, IL-1β	[103]
Fucoidan	12.5–25–50 μg/mL	*Turbinaria ornata*	Zebrafish embryos—LPS induced inflammation In vitro: RAW 264.7—LPS induced inflammation	1,2	↓ NO, ROS production ↑ Cell viability dose dependent activity In vitro: ↓ iNOS, COX-2 ↓ NO, PGE2 ↓ TNF-α, IL-1β	[53]
Fucoidan	12.5–25–50 μg/mL	*Laminaria japonica*	Zebrafish embryos—LPS induced inflammation In vitro: RAW 264.7—LPS induced inflammation	1,2	↓ NO, ROS production ↓ cell death rate dose dependent activity In vitro: ↓ MAPK, NF-κβ ↓ NO, ↓ TNF-α, IL-1β, IL-6	[54]
Laminarin, fucoidan and ash	Laminarin 1 g, fucoidan 0.8 g, day 107 of gestation until weaning (day 26)	*Laminaria*	Pregnant + Lactating sows	2	↑ Colostrum IgA ↑ Piglets serum IgG	[64]
Fucoidan	6 g/kg feed for 21 days	*Laminaria japonica*	Immunosuppressed African catfish	1,2	Macrophages: ↑ oxidative burst, ↑ phagocytic activity Lymphocytes: ↑ transformation index Serum: ↑ lysozyme, NO and bactericidal activity ↑ Survival rate in challenge test	[70]
Fucoidan-rich extract	2% inclusion, 45 days feeding trial	*Sargassum wightii*	Sutchi Catfish	1,2	Macrophages: ↑ oxidative burst, ↑ phagocytic activity ↑ Total lymphocyte count ↓ Albumin/Globulin ratio ↑ IFN-γ ↑ Survival rate in challenge test	[73]
Laminarin	0.2 g/kg/day for 21 days	*Laminaria digitata*	Rainbow trout	1,2	↑ TNF-α, IL-8	[72]
Fucoidan	0.05%, 0.1% and 0.2% per kilogram feed	*Sargassum horneri*	Yellow catfish	1,2	Macrophages: ↑ oxidative burst, ↑ phagocytic activity Serum: ↑ lysozyme ↑ CAT ↑ SOD, ↓ MDA↑ Survival rate in challenge test	[75]
Fucoidan-rich seaweed extract (FRSE) + Methionine	2% FRSE + 0.3 methionine	*Sargassum wightii*	Carp (*Labeo rohita*)	1,2	↑ respiratory burst activity, phagocytic activity, ↑ MPO activity, lysozyme activity, ↑ total immunoglobulin and TLC	[71]
Laminarin	0.5–1.0% inclusion in diet for 48 days	Commercially sourced	Grouper (*Epinephelus coioides*)	1,2	↑ IL-1β, IL-8 and TLR2 ↑ lysozyme, CAT and SOD ↑ growth rate and the feed efficiency	[76]
Fucoidan	250–500 mg/kg feed	*Sargassum*, *Padina* and *Turbinaria*	White shrimp (*Litopenaeus vannamei*)	1,2	↑ THC, PA and SOD ↑ LGBP, Toll and lectin	[74]
Laminarin, fucoidan and ash	Lam 1 g, Fuc 0.8 g, d 83 of gestation until weaning (day 28)	*Laminaria*	Pregnant + Lactating sows	1,2	↑ Piglets villus height in the jejunum and ileum	[67]
Extract	D 83 of gestation until weaning (day 28)	*Ascophyllum nodosum* and *Fucus*	Pregnant + Lactating sows	1,2	↑ Piglets CD4+ and CD8+ T cells	[66]
Laminarin	1 g/day Pre-weaning (0–62 days) and post-weaning (63–93 days)	*Laminaria*	Holstein Friesian bull calves	1,2	↓ Growth ↑ serum haptoglobin ↓ lymphocyte levels ↓ stimulated IFN-γ (in vitro challenges)	[68]
Sodium alginate oligosaccharides	0, 0.04 and 0.2% of diet	Unknown	broiler chickens Salmonella Enteritis challenge	1,2	in unchallenged animals: ↑ IFN-γ, IL-10, IL-1β	[69]
Highly viscous polysaccharide extract (HVPE)	10–30–100 mg/kg/day for 14 days	*Kjellmania crassifolia*	C57BL/6 mice	1,2	ConA-stimulated spleen cells: ↑ IFN-γ, IL-12, IL-6, IgA secretion ConA-stimulated Peyer’s patch cells: ↑ IgA ↑ Peritoneal macrophage phagocytic activity	[77]
Fucoidan (LMWF)	200–400–1000 mg/kg/day (6 weeks)	*Laminaria japonica*	Sprague Dawley rats, Mycoplasma pneumoniae antigen stimulation	1,2	↑ Spleen weight, splenocyte proliferation potential ↑ NK cell activity, ↑ Phagocytic activity ↑ IFN-γ, IL-2 IL-4 ↑ IgG, IgA, ↓ IgE ↑ Antigen-specific antibodies	[61]
Fucoidan containing product (88.3% purity)	410–1025 mg/kg	*Cladosiphon okamuramus*	Balb/c mice, OVA-immunized	1,2	↑ Immune cell proliferation, IL-2, ↑ macrophage phagocytic activity ↑ serum IgM, IgG, IgA ↓ IgE ↓ IL-4, -5	[62]
Fucoidan (LMWF, MMWF, HMWF)	Unclear	*Cladosiphon okamuranus*	Pathogen free Male Balb/c mice	1,2	HMWF: ↑ Proportion CD8+ T-cells in spleen, ↓ CD4/CD8 ratio	[78]
water-soluble polysaccharides	50–100–200 for 10 days	*Sargassum fusiforme*	Cyclophosmamide-immunusuppressed ICR mice	1,2	Compared to the cy model group: ↑ spleen index ↑ splenic lymphocyte proliferation potential ↑ splenocyte produced IL-2, Il-6, IFN-γPeritoneal macrophages: ↑ phagocytic activity, ↑ produced IL-2, Il-6, IFN-γ	[92]
Fucoidan	150 mg/kg daily for 2 weeks	*Fucus vesiculosus*	mice	1,2	increased cytolytic activity of NK cells	[89]
Laminarin	500–1000 mg/kg/day for 10 days	Unknown, commercially sourced	Cyclophosmamide-immunusuppressed male Balb/c mice	1,2	Compared to the cy model group: ↑ cytotoxicity of NK cells ↑ serum IL-12 and IFN-γ	[94]
Fucoidan	0.1–0.5 mg/day for 14 days	*Undaria pinnatifada*	HSV-1 infected mice/5 fluorouracil immunosuppressed mice	2	Protection against herpes infection ↑ Macrophage NO production ↑ CTL activity ↑ B-cell blastogenesis↑ Antibody titers	[82]
Fucoidan	25–250 mg/kg/day, 3 times weekly for 4 weeks	Unknown	Leishmania infection, Balb/c mice	3	In vitro: 93% reduction Amastigote multiplication in macrophagesIn vivo: complete elimination parasite in liver and spleen Th2 ◊ Th1 response Splenocytes: ↑ superoxide and NO production	[79]
Fucoidan	500 mg/kg every 2 days, for 6 weeks	*Fucus vesiculosus*	Schistosomiasis Japonica infeced C57BL/6 mice	3	↓ Granuloma size, ↓ hepatic inflammation↓ IL-6, IL-12, TNF-α ↑ IL-4, IL-13 ↑ IL-10, TGF-β ↑ Th2 response ↑ Treg infiltration in liver	[106]
Fucoidan	0.034 g/mouse/day 10 days before, 40 days after tumor inoculation	*Undaria pinnatifada*	A20-tumor bearing Male Balb/c mice/Do-11-10-Tg mice (transgenic for TCR)	3	↑ NK cell activity ↑ CTL activity ↑ IFN-γ, IL-12	[84]
Ascophyllan	500 mg/kg. 4 days before–10 days after tumor implantation	*Ascophyllum nodosum*	S-180 sarcoma bearing SPF male ddY mice	3	Oral route stronger anti-tumor effects than intraperitoneal injection, effects via interaction with intestinal immune system ↓ 69% tumor size reduction ↑ TNF-α, IL-12	[91]
Polysaccharides	100–200 mg/kg for 28 days	*Sargassum fusiforme*	A549 adenolungcarcinoma- bearing Balb/c nuce mice	3	↑ Serum TNF-α ↑ Peritoneal macrophage production of TNF-α, IL-1β ↑ Splenocyte lymphocyte proliferation Liver and Kidney: ↑ SOD, ↓ MDA	[87]
Fucoidan (LMWF/IMWF/HMWF)	5 g/kg/day	*Cladosiphon okamuramus*	Colon 26 tumor bearing mice; Myd-88^−/−^ mice	3	No intestinal absorption Effects via interaction with intestinal immune system All: ↑ Median survival timeIMWF ↓ Tumor weight, ↓ Cell divisions, ↑ Apoptosis HMWF: ↑ Splenic NK cell numbers	[85]
Polysaccharide	100–200–400 mg/kg for 28 days	*Sargassum fusiforme*	HepG2-tumor-bearing mice	3	↑ Serum TNF-α, NO, IL-1β, IgM ↑ Peritoneal macrophage production of TNF-α, IL-1β ↑ Apoptosis in HepG2-tumor cells	[86]
Polysaccharides	50–100–200 mg/kg	*Sargassum fusiforme*	CNE- tumor- bearing mice	3	↑ Serum NO, IL-1β, IgM ↑ Peritoneal macrophage production of NO, IL-1β↑ Splenocyte lymphocyte proliferation ↑ IgM production Activity is, in part, mediated via TLR2 and TLR4	[88]
Fucoidan	200–400 mg/kg (6×/week) for 4 months	*Fucus vesiculosus*	DMBA-induced mammary carcinogenesis in female Sprague Dawley rats	3	↓ Tumor incidence, ↓ Tumor weight ↑ Serum IL-6, IL-12p40, IFN-γ ↓ IL-10 TGF-β ↓ MRNA expression levels of FOXP3, TGF-β, ↑ IFN-γ in tumors ↓ Foxp3, PD1, =PDL1/PDL2 protein levels in tumors ↓ p-PI3K, p-AKT protein levels in tumors	[90]
Fucoidan	5 mg/day for 10 days (start direct after infection)	*Undaria pinnatifada*	Influenza infected mice	3	↑ Survival ↑ Inhibition of viral replication ↑ Mucosal antibody levels (IgA) ↑ Serum antibody titer (IgM, IgG)	[80]
Fucoidan	6 g/day for 6–13 months	Unknown, commercially obtained	Human, HTLV-infected patients	3	↓ HTLV proviral load =CD4^+^, CD8^+^ frequencies =NK-cell, mDC and pDC frequencies	[81]
Oligo Fucoidan	4400 mg/day for 48 weeks	Unknown, commercially sourced	Patients with chronic Hepatitis B virus 2193	3	vitamin D dependent activity ↓ HBV DNA ↑ CD4^+^CD45RO^+^ and CD8^+^CD45RO^+^	[109]
Fucoidan	3 g/day for 6 months	*Cladosiphon okamuranus*	Human, male cancer survivors	3	↑ NK-cell activity	[165]
Fucoidan	100–1000 mg/day for 4 weeks	*Fucus vesiculosus* (85%), *Macrocystis pyrifera* (10%), *Laminaria japonica* (5%)	Human volunteers	3	↑ Cytotoxic T cell numbers ↑ Phagocytic capacity monocytes ↓ IL-6No dose response	[110]
Fudoidan (75% pure)	3 g/day for 12 days	*Undaria pinnatifada*	Human volunteers	3	↑ CD34+ cells, CD34+ cells expressing CXCR4 (45% ◊ 90%)↑ serum SDF-1 and IFN-γ	[166]
Fucoidan	300 mg/day for 4 weeks	*Mekabu fucoidan*	Human, Influenza vaccinated elderly	3	↑ Antibody titers	[167]
Sulphated polysaccharide	20–40–80 mg/kg/day for 5 days	*Sargassum hemiphyllum*	Mouse–arachidonic acid induced ear-inflammation	2	dose dependent activity ↓ Ear swelling and erythema ↓ TNF-α, IL-1β, IL-6 ↓ neutrophil infiltration	[95]
Combined preparation including fucoidan	Fucoidan: 18–54 mg/kg/day for 7 days	*Laminaria japonica*	Mouse–Carrageenan induced pouch inflammation	2	↓ NO, PGE2 ↓ neutrophil and macrophage recruitment	[96]
Fucoidan	0.02 g/kg for 14 days	Commercially sourced from Sigma	Mouse–aspirin induced ulcer	2,3	Protection against ulceration ↓ AST, ALT ↓ PGE2 ↓ IFN-γ, IL-6, IL-10 ↓ stomach glycogen	[97]
Fucoidan	0.05% *w*/*w* in chow	*Cladosiphon okamuranus*	Mouse–DSS-induced colitis	2	↓ IFN-γ, IL-6 ↑ IL-10, TGF-β	[98]
Fucoidan & Fucoidan-polyphenol complex (Synergy)	10 mg/mouse, 400 mg/kg/day	*Fucus vesiculosus*	Mouse–DSS-induced colitis	2,3	Oral synergy best results ↑ colon length ↓ colon weight/body weight, spleen weight ↓ Histology damage score↓ TNF-α, IL-12 IP fucoidan actually worsened colitis symptoms	[99]
Fucoidan	200 mg/kg	*Laminaria japonica*	Mouse–myocardial ischemia-reperfusion injury	2,3	↓ myocardial infarct size ↓ HMGB1, p-IκB-a and NF-κB ↓ TNF-a and IL-6, ↑ IL-10 ↓ infiltration PMNs, ↓ MPO activity ↓ histopathological damages in myocardium	[104]
Fucoidan	200 mg/kg/day for 14 days	*Sargassum hemiphyllum*	C57BL/6 mice, irradiation induced pneumonitis and lung fibrosis	3	↓ Lung fibrosis ↓ Neutrophil and macrophage infiltration ↓ TIMP-1, CXCL-1, MCP-1, MIP-2, IL-Ra ↓ Procollagen-1α	[105]
Fucoidan (LMWF: 1 ± 0.2 kDa MMWF: 3.5 ± 0.3 kDa HMWF: 100 ± 4 kDa)	300 mg/kg dissolved in water, one day after booster immunization until the end of the experiment (day 22–47)	*Undaria pinnatifada*	Male DBA/1J mice, Collagen-induced arthritis	2,3	LMWF: ↓ reduced severity of inflammation↓ IFN-γ, TNF-α ↓ IgG2a, HMWF: ↑ Increased severity of inflammation↑ IFN-γ, TNF-α ↑ IgG2a ↑ adhesion molecules and migration potential of macrophages	[102]
Fucoidan	4 g/day for 4 at least weeks	*Cladosiphon novae Caledoniae*	Advanced cancer patients	3	Responsiveness of IL-1β independent prognostic factor for QOL-scores ↓ TNF-α, IL-1β, IL-6 = QOL-scores	[111]
Fucoidan	400 mg/kg for 22 weeks	*Fucus vesiculosus*	Mice, colorectal carcinogenesis model	2,3	↑ Beneficial Microbiome modulation ↓ IL-17 and IL-23 ↑ IFN-γ, IL-4 and IL-10 ↑ NK cells, CD4+ T cells in blood	[101]
Fucoidan	300–600 mg/kg/day for 5 weeks	*Fucus vesiculosus*	Non-obese diabetic mice (autoimmune diabetes model)	2	↑ serum insulin levels Delayed onset and ↓ incidence of diabetes ↓ Th1 cytokines ↑ Th2 cytokines Dendritic cells: ↓ MHC-II, ↓ CD86 Pancreatic cells: ↓ TLR4 expression Microbiota: ↑ Akkermansia, ↑ Lactobaccillus, ↓ Bacteroides	[108]
Fucoidan	400 mg/kg	*Ascophyllum nodosum*	Colonic inflammation- induced SPF- C57BL/6 mice (antibiotics treated)	3	↓ Colonic inflammation↓ Dysbiosis ↓ Infiltration of inflammatory cells ↓ Space between mucosa and submucosa ↑ Crypt depth ↓ TNF-α, IL-1β, IL-6, ↑ IL-10	[100]
Fucoidan	200 mg/kg	*Laminaria japonica/Ascophyllum nodosum*	SPF C57BL/6 J mice, High fat diet	3	↓ Body weight gain, ↓ Fat mass ↓ Insulin resistance ↓ Endotoxemia (↓ LBP) ↓ Systemic inflammation (↓ TNF-α, IL-1β, MCP-1) Microbiome: ↑ Akkermansia, Alloprevotella, Blautia, Bacteroides	[47]
Fucoidan and Fucoxanthin	275 mg LMF	Unknown, commercially obtained	Clinical trial, NAFLD patients		↓ AST =adiponectin	[168]
Fucoidan	50 mg/kg for 14 days	*Fucus vesiculosus*	Balb/C mice, ConA-induced acute liver injury	3	↓ AST, ALT ↓ Histopathological changes ↓ IFN-γ, TNF-α ↓ Apoptosis inhibition	[107]
Fucoidan	1 or 5% inclusion in diet (12 weeks)	*Cladosiphon okamuranus*	Diet-induced dyslipidemia in ApoE^−/−^ mice	3	↓ Tissue weight (liver and WAT), hepatic steatosis ↓ Blood lipids (TC, TG, non-HDL-C)↓ Blood glucose ↑ Plasma LPL activity, HDL-C ↑ Insulin-sensitivity ↑ Pparα, ↓ Srebf1	[169]
LJP12	50–100–200 mg/kg/day	*Laminaria japonica*	Atherogenic diet fed LDLR^−/−^ mice	3	Dose-dependent activity ↓ Atherosclerotic plaque formation ↓ Plasma lipids (TC, TG, LDL-C), ↑ HDL-C/LDL-C ↓ Systemic inflammation (↓ TNF-α, IL-1β, IL-6, MCP-1, ↑ IL-10) ↑ SOD, ↓ MDA ↓ p-p 65, p-Iκβ, p-ERK, p-JNK, p-P38	[112]
Fucoidan (LMWF)	200 mg/kg/day	*Laminaria japonica*	Diet-induced dyslipidemia in ApoE^−/−^ mice	3	↓ TG, OX-LDL ↓ p-JNK, cyclin-D1 ↓ IL-6, ↑ IL-10 ↓ Macrophages ◊ foam cells ↓ Migration of SMCs into intimal layer	[114]
Fucoidan	Unknown	*Fucus vesiculosus*	Rats	3	↓ Endothelial hyperplasia, ↓ vascular modulation ↓ α-actin^+^ cells ↓ Vessel inflammation↓ Macrophage infiltration ↓ Apoptosis in vessel wall	[115]
Fucoidan (LMWF)	200 mg/kg/day	*Laminaria japonica*	Diet-induced dyslipidemia in ApoE^−/−^ mice	3	↓ Inflammatory infiltration ↓ Limit enlargement of AAA ↓ Maximal aortic diameter Preserved elastin ↓ TNF-α, IL-1β, MCP-1 ↓ MMP	[116]
Fucoidan	50–100 mg/kg/day	*Laminaria japonica*	In vitro: oxLDL treatment RAW264.7 In vivo: Atherogenic diet fed LDLR^−/−^ mice	3	In vitro: ↓ oxLDL-induced LOX-1, ↓ TNF-α, IL-1β, IL-6 ↓ ICAM-1, VCAM-1 ↓ ROS In vivo: ↓ Atherosclerotic plaque formation ↑ Plaque stability↓ Macrophage infiltration ↓ Plasma lipids (TC, TG, non-HDL-C) ↓ ROS	[113]
Fucoidan (LMWF) <3 kDa	5 mg/day. With or without added probiotics	*Undaria pinnatifada*	Male Balb/c mice.	2	Fucoidan enhances effects of probiotic strain ↑ IFN-γ, TNF-α, IL-6, IL-12 OVA-immunization: ↓ lgE, IL-4, ↑ IFN-γ Desulphated Fucoidan no effects	[170]
Extracted polysaccharide (not fucoidan)	50 mg/kg/day for 2 weeks	*Laminaria japonica*	SPF Kunming mice, OVA-immunized asthma model	2,3	↓ Eosinophils in BALF ↓ lung inflammation↓ Serum IgE ↑ IL-12, ↓ TGF-β, IL-13 in BALF and lung	Lin et al. 2015. Multidiscip Respir Med. PMID: 26110056 [58]
Fucoidans	100–400 mg/kg/day	*Undaria pinnatifada*	OVA-immunized female Balb/c mice, exacerbated allergic astma	2,3	↓ Infiltration of macrophages, neutrophils, CD4^+^ lymphocytes ↓ Lipid peroxidation ↓ IL-4, ↓ IgE ↓ Mast cell activation, degranulation ↓ Mucus hypersecretion, ↓ Goblet cell hyperplasia	Herath et al. 2020. Molecules. PMID: 32580518 [57]
Fucoidan	100–400 μg/mouse/day for 4 days	*Laminaria japonica*	Female Balb/c mice, passive cutaneous anaphylaxis model	2,3	↓ allergic symptoms (Edema) Oral fucoidan anti-allergy effects, IP-fucoidan no effects =Serum IgE, IgG1 ↑ Galectin-9 secretion by intestinal epithelial cells	[59]
Fucoidan	60 μg/mouse/day for 17 days after OVA-immunization	*Laminaria japonica*	Female Balb/c mice, OVA-immunized	2,3	↓ allergic symptoms (Rectal temperature reduction) =Serum IgE ↑ Serum galectin-9 ↓ Degranulation of mast cells ↓ IgE-attachment on mast cells	[60]
Alginate, 108 kDa	2 mg alginate one day before every OVA-challenge	*Laminaria japonica*	OVA-immunized female Balb/c mice, food allergy model	2,3	↓ IL-4, ↓ IgE ↓ Mast cell degranulation, ↓ Histamine ↑ Number of Tregs in spleen ↓ Th0 ◊ Th2	[63]

^1^ Phases of inflammation as indicated in Table 1: 1-initiation of inflammation; 2-short term amplification and regulation; 3-short- or long-term consequences. ^2^ Symbols used: ↑—increase; ↓—decrease; =—no change.

## Appendix C

**Table A3:** Effects of oral supplementation of brown seaweed derived phytosterols on inflammation-related parameters.

Compound/Format	Effective Concentrations	Algal Source	Experimental Model	Inflammatory Phase ^1^	Significant Findings ^2^	Reference
Seaweed extract containing fucosterol Enzyme-modified extract	NH: 200 mg/kg EH: 50, 100 and 200 mg/kg; 3 weeks	*Sargassum fusiforme = Hijiki*	Male C57BL/6 mice; ConA activation induced splenocyte proliferation	1	↑ splenocyte proliferation In peritoneal Macrophages:↓ IL-6 ↓ IL-1β ↓ TNF-α (EH > NH)	[22]
Lipid extract containing 24(S)-Saringo-sterol	50% (*w*/*w*), 10 weeks	*Sargassum fusiforme*	APPswePS1ΔE9 Mice, Alzheimer’s disease +/− seaweed supplement	3	↑ LXR-responsive gene expression in CNS ↑ ApoE	[118]
Fucosterol extract	25, 50 or 100 mg/kg; 3 days	*Eisenia bicyclis*	Male BALB/c mice, ConA- induced acute liver injury after Fucosterol pretreatment	1	↓ TNF-a ↓ IL-6 ↓ IL-1β ↓ NF-κB p65 ↑ PPARγ expression ↑ p-P38 MAPK levels	[117]
ethyl acetate extract => Fucosterol	200 mg/kg 2 weeks	*Sargassum fusiforme*	NC/N ga male mice, DNCB induced AD-like dermatitis	allergy	↓ Scratching ↓ epidermal thickness of dorsal skin ↓ mast cells ↓ serum level of IgE In cultured splenocytes: ↓ IL-4 ↓ TNF-α ↑ IFN-γ	[171]

^1^ Phases of inflammation as indicated in Table 1: 1-initiation of inflammation; 2-short term amplification and regulation; 3-short- or long-term consequences; ^2^ Symbols used: ↑—increase; ↓—decrease; =—no change.

## Appendix D

**Table A4:** Effects of oral supplementation of brown seaweed derived phenolic compounds on inflammation-related parameters.

Compound/Format	Effective Concentrations	Algal Source	Experimental Model	Inflammatory Phase ^1^	Significant Findings ^2^	Reference
(poly)phenol-rich seaweed extract	400 mg; 8 weeks	*Ascophyllum nodosum*	80 participants; 30–65 years old with a body mass index (in kg/m^2^) ≥ 25.	Steady state	↓ DNA damage in obese people No significant differences in CRP, antioxidant status and inflammatory cytokines	[7]
MeOH–EtAc extract => phlorotannin	100–200 mg/kg/day 3 days before experiment	*Eisenia bicyclis = Ecklonia bicyclis*	Sprague Dawley rat, platelet aggregation + activation	Steady state	Eisenia bicyclis extract strongly inhibits in vivo platelet aggregation. Mechanism in vitro: downregulated ADP-induced platelet activation (Ca-mobilization, fibrinogen binding, granule release—mediated via decreased Src, PI3K, PLCgamma2, MAPK signaling)	[122]
MeOH extract=> Eckol	25, 50, 100 μg/mL, pretreatment 2 h prior to anaphylaxis induction 25, 50, 100 μg/mL, 24 h	*Ecklonia cava*	BALB/c mice; anti-DNP-IgE induced PCA C57BL/6 male mice; anti-DNP-IgE and IgE-BSA induced allergic	allergy	↑ binding of IgE to FcɛRI ↓ FcɛRI expression ↓ mRNA level of IL-1β, IL-6, TNF-α, IFN-γ ↓ NF-κB nuclear translocation ↓ IκB degradation ↓ secretion of inflammatory mediators, such as histamine, β-hexosaminidase, leukotrienes and prostaglandins ↓ production of Th2-type cytokines, such as IL-4, IL-5, IL-13 (not 25 ug/mL)	[123]
MeOH-ethyl acetate extract (crude (72–74%) phlorotannins = phloroglucinol oligomer);	oral 1× or 2×; 0.1, 1 mg/ear	*Ecklonia kurome* *Ecklonia arborea*	male ICR mouse; AA induced ear oedema	allergy	inhibitory effects are comparable to known anti-allergic agents presumed mechanisms: inhibition of MC degranulation + inhibition of COX-2 and LOX, and to lesser extent PLA2, activities	[125]
(poly)phenol-rich brown algae extract	100 mg/kg 500 mg/kg; 12 weeks	*Ecklonia cava*	C57BL/6 male mice; HFD+/− seaweed supplement	1,3	↓ MCP1 ↓ TNF-α ↓ IL-1 ↓ COX-2 (slightly decreased) protein level ↓ NF-κB protein level↓ Body weight ↓ adipose tissue weight ↓ deposition in the liver ↓ TG and TC levels ↓ leptin and adiponectin ratio ↓ Glucose Tolerance Test ↓ Insulin Resistance ↑ protein levels of P-AMPK ↑ SIRT1 protein levels ↑ PGC1α protein levels in the nucleus	[128]
A single compound Pyrogallol-Phloroglucinol-6,6-Bieckol from the brown algae ethanoic extract	2 mg/kg; 4 weeks	*Ecklonia cava*	C57Bl/6N mice; HFD ^+/−^ seaweed supplement	1,2,3	In visceral fat: ↓ TNF-a mRNA levels ↓ IL-1β mRNA levels ↓ Macrophage infiltration ↓ M1/M2 ratio (CD86 and CD80 lower, CD163 and CD206 higher) ↓ RAGE ↓ RAGE-RAGE Ligand Bonding ↓ Body weight ↓ size of visceral adipocytes ↓ fat mass ↓ serum TG and TC ↓ AGEs, HMGB1 and S100beta secret by adipocytes	[137]
Polyphenols-rich Ethanol extract	200, 400 mg/kg; 7 days	*Sargassum horneri*	BALB/c female mice; PM induced airway inflammation in allergic asthma	1,2	↓ granulocyte infiltration ↓ macrophage infiltration ↓ TLRs expression in lungs ↓ NF-κB pathway activation ↓ pro-inflammatory cytokines IL-1β, TNF-α, IL-6 expression in lung↑ IL-10 expression in lung ↓ IL-25, IL-33, IL-8, TGF-β in lung ↓ cytokines (IL-25, IL-33) in serum	[124]
phlorotannins-rich extract	PREC (75 and 150) or dieckol (50 and 100 mg/kg; 4 weeks Combination cisplatin (1, 3 or 5 μM) with PREC (35, 50, 75 or 100 μg/mL); 48 h	*Ecklonia cava*	BALB/c athymic female nude mice; SKOV3 cells induced ovarian carcinoma	1	PREC: ↓ Akt in ovarian cancer ↓ NF-κB in ovarian cancer ↑ intracellular ROS in ovarian cancer ↓ cisplatin-induced ROS in normal HEK293 ↑ ROS production in SKOV3 cells the combined treatment of PREC and cisplatin: ↓ ROS production in in SKOV3 cells and normal HEK 293cells Dieckol: ↑pathwayscisplatin-induced ROS production Dieckol: ↓pathwayscisplatin-induced ROS production (slightly)	[129]
phlorotannin derived from the brown alga	0.5 and 1.0 mg/kg/day; Pretreatment for 7 days	*Ecklonia stolonifera*	Male Kunming mice; CCl4 induced acute liver injury	1,2	↓ TNF-a ↓ IL-1β ↓ IL-6 ↓ ROS ↑ IL-10 ↑ CD11c+	[135]
Ethyl acetate fraction containing diphlorethohydroxycarmalol	6.25, 12.5, 25 μg/mL	*Ishige okamurae*	zebrafish embryo; FD induced ROS, NO production and cell death	1,2	↓ ROS production ↓ NO production	[134]
polyphenol-rich brown algae extract	25, 50 and 100 μg/mL; pretreatment for 1 h	*Ecklonia cava*	Zebrafish embryos	1,2	↓ ROS expression ↓ NO expression ↓ iNOS	[133]
chloroform–methanol extract Apo-9′-fucoxanthinone (AF)	25, 50 and 100 μg/mL; pretreatment for 1 h	*Sargassum muticum*	Zebrafish embryos	1,2	↓ IL-1β, ↓ TNF-α ↓ ROS ↓ COX-2 ↓ NF-κB ↓ MAPK ↓ iNOS ↓ NO	[131]
Six purified phlorotannins ((eckol; 6,60-bieckol; 6,80-bieckol; 8,80-bieckol; phlorofucofuroeckol (PFF)-A and PFF-B)	10, 100, 200 µM	*Eisenia arborea*	ICR mice; AA, TPA and OXA induced ear swelling	1	↓ COX-2 mRNA expression ↓ COX-2 enzymatic activity	[136]
Ethanol extract including Dieckol, 2,7-phloroglucinol-6,6-bieckol (PHB), PFF-A and pyrogallol-phloroglucinol-6,6′-bieckol (PPB)	2.5 mg/kg; 4 weeks	*Ecklonia cava*	57BL/6N mice; HFD^+/−^ seaweed supplement	1,2,3	↓ TNF-α ↓ IL-6 ↓ TLR4 expression ↓ NF-kB expression ↓ CD11b ↓ CD86 ↑ CD206 ↓ body weight ↓ food intake ↓ fat mass ↓ leptin resistance ↑ leptin sensitivity ↓ PPAR expression ↓ CEBP expression ↓ FAS expression ↓ ACC expression ↓ ER	[126]
Ethanolic extract including phlorotannin	70 mg/kg; 4 weeks	*Ecklonia cava*	male 57BL/6N mice, HFD^+/−^ seaweed supplement	1,2,3	↓ NF-kB ↓ TNF-α ↓ IL-6 ↓ TLR4 expression ↓ CD4 and CD8a ↓ CD11b ↑ CD206 ↓ CD80 ↑ IL-10 ↑ TGF-β mRNA ↓ Body weight ↓ Food intake ↓ Adipocyte size ↓ Leptin resistance ↓ RAGE	[127]
phlorotannins-rich ethanolic extract	100, 200, 400 mg/kg/day; 8 weeks	*Ecklonia cava*	Male Sprague Dawley rats; Experimental periodontitis induced by placing a sterile 4-0 silk ligature around the gingival cervix of the right mandibular second molar teeth	1,2	↓ COX-2 activity ↓ PGE2 ↓ NO ↓ iNOS	[130]
Ethanolic extract and PPB	ECE: 70 mg/kg PPB: 2.5 mg/kg; 4 weeks	*Ecklonia cava*	Male C57BL/6N mice; HFD^+/−^ seaweed supplement	1,2,3	↓ MCP-1 ↓ TNF-α ↓ IL-6 ↓ IL-10 ↓ iNOS ↓ CD80 ↑ CD206 ↓ ER stress ↓ size of white adipocytes ↑ PPAR expression	[138]
baicalein, luteolin, rosmarinic acid and cis-2-decenoic acid (10-HAD)	Test diet for mice:(baicalein (650 mg/kg diet), luteolin (300 mg/kg diet), rosmarinic acid (500 mg/kg diet), monolaurin (500 mg/kg diet), 10-HAD (500 mg/kg diet)); 4 weeks Capsule for human: (baicalein 250 mg/day, luteolin 75 mg/day, rosmarinic acid 100 mg/day, monolaurin 250 mg/day, 10-HAD 100 mg/day and iodine 0.15 mg/day); 3 times/day 6 months	Unknown	C3H/HeN inbred female mice; LD infection ^+/−^ test diet Adult human; with a history of acute LD	1,2	Animal study: ↓ IL-6 ↓ TNF-α ↓ INF-γ Human study: By administration of the composition, 17.7% had slight physical and psychological improvement, and 17.7% were none responsive ↓ IL-17	[139]
commercial (30%) polyphenolic fraction (VNP)oral admin	Rat: 1 dose 2000 mg/kg Human: 2400 mg/day 8 weeks	*Sargassum fusiformi*, *Ecklonia kurome*, *Ecklonia Stolonifera*, *Eisenia bicyclis* and *Ecklonia cava*	Sprague Dawley rat human (men)	1	rat: serum ferric reducing antioxidant power (FRAP) significantly elevated 30 min after treatment, but declined quickly thereafterhuman: ↑ erectile function (significantly) =usefulness of these polyphenolic compounds as chemo preventive agents against vascular risk factors originating from oxidative stress	[132]
Compound 21	35 mg/kg; 7 days	Synthesized by the department of pharmaceutical chemistry	Female Lewis rats; MBP-induced EAE	3	Attenuates neurological deficits, immune infiltration and demyelination in EAE rats Reduces the population of Th1/Th17 cells and inhibits their infiltration into the CNS	[142]
phloroglucinol derivative Compound 21	1, 5, 10, 20, 50 mg/kg; 4 weeks	Unknown	Cuprizone induced intoxication in mice,	3	significantly improved neurological dysfunction and motor coordination impairment decreased microglia and astrocytes activities and the subsequent neuro-inflammatory response	[141]
EtOAc Fraction of seaweed Crude Extract	200 mg/kg; 8 weeks	*Ecklonia* cava from Jeju or Gijang	C57BL/6 mice; HFD+/− seaweed supplement	1,3	↓ TNF-a mRNA levels ↓ IL-1ß mRNA levels↓ body weights ↓ weight gain ↓ fat tissue mass ↓ Plasma ALT and cholesterol levels ↑ PPARγ2 mRNA expression ↑ C/EBPα, mRNA ↓ Blood glucose levels	[140]

^1^ Phases of inflammation as indicated in Table 1: 1-initiation of inflammation; 2-short term amplification and regulation; 3-short- or long-term consequences; ^2^ Symbols used: ↑—increase; ↓—decrease; =—no change.

## Appendix E

**Table A5:** Effects of oral supplementation of brown seaweed derived Fucoxanthin(ol) and Meroterpenoid on inflammation-related parameters.

Compound/Format	Effective Concentrations	Algal Source	Experimental Model	Inflammatory Phase ^1^	Significant Findings ^2^	Reference
Fucoxanthin-containing extract	13, 26 and 65 mg/kg Fx for 4 weeks	*Laminaria japonica*	Male Sprague Dawley rats (5 weeks old; ±200 g), diabetes induced by i.p. administration of streptozotocin followed by nicotinamide	1,3	↓ TNF-α mRNA expression in plasma and testis ↓ IL-6 mRNA expression in plasma and testis ↑ CAT, SOD and GPx activity in plasma ↑ SOD activity in testis ↓ H_2_O_2_ and O_2_^−^ levels ↓ MDA level in plasma, testis and sperm ↓ Plasma glucose level ↓ Insulin level ↓ HOMA-IR ↓ SOCS-3 mRNA expression in hypothalamus	[150]
Fucoxanthin-rich brown algae extract	Colitis: 1, 2 or 5 g/kg bw/day for 7 days; CACC: 0.5, 1 or 2.5 g/kg bw/day for 11 weeks	*Sargassum muticum*	Male BALB/c mice (6–8 weeks old), 3% DSS-induced colitis for 14 days or CACC induction by a single i.p. injection of azoxymethane + 2% DSS for 7 consecutive days at weeks 3, 6 and 9	1,2	DSS-induced colitis: ↓ MDA level in colonic tissue=MDA level in plasma ↑ SOD levels in plasma ↓ TNF-α in colonic tissue and plasma ↓ IL-6 in plasma =IL-6 in colonic tissue ↓ Total NO content in colonic tissue↓ NO release in colonic tissueCACC: ↓ MDA level in colonic tissue=MDA level in plasma ↑ SOD levels in plasma ↓ TNF-α in colonic tissue and plasma =IL-6 in colonic tissue and plasma ↑ proliferation T and B cells ↓ Total NO content in colonic tissue↓ NO release in colonic tissue	[151]
Fucoxanthin-rich extract	100, 200 or 500 mg/kg Fx, dissolved in olive oil and administered for 5 days	*Sargassum glaucescens*	Male Syrian hamsters (7 weeks old; 80–90 g), Cisplatin induced testicular damage group with CP i.p. injection before Fx treatment	1,2,3	↑ CAT and GPx activity in testis ↑ SOD activity in plasma ↓ MDA level in testicular tissue, sperm and plasma ↓ NO levels =Body weight	[152]
Fucoxanthin	0.4% and 0.6% Fx for 5 weeks	Unknown	Kunming strain mice (20–22 g) fed regular chow or HFD for 9 weeks + 5 weeks with Fx added to diet	1,2,3	↓ Mammary gland inflammation↓ MPO activity ↓ IL-1β in the blood ↓ TNF-α in the blood ↓ MDA level ↓ COX-2 and iNOS mRNA expression ↓ Body weight gain	[155]
Fucoxanthin-rich wakame lipids (WLs)	WLs with 1.06% Fx; WLs with 2.22% Fx, administered for 5 weeks	*Undaria pinnatifida*	Male C57BL/6J mice (8 weeks old), normal-fat or HFD for 10 weeks followed by normal-fat or HFD with Fx for 5 weeks	2,3	↓ MCP-1 mRNA expression in WAT =TNF-alfa mRNA expression in WAT ↓ Body weight gain =Food intake ↓ WAT weight gain ↓ LDL cholesterol ↓ Plasma leptin ↓ Plasma insulin ↓ blood glucose ↓ Leptin mRNA expression in WAT ↑ ADRB3 mRNA expression in WAT ↑ GLUT4 mRNA expression in skeletal muscle	[156]
Fucoxanthin and fucoxanthinol	150 nmol/mouse, before induction of ear swelling	Unknown	Male ICR-strain mice (4 weeks old), AA-, TPA- or OXA-induced ear swelling	1	↓ Inflammation in mouse ear swelling ↓ PLA2 and COX-2 enzymatic activities	[157]
Lipid extracts containing fucoxanthin	0.10% Fx, 1 g Fx/kg of diet for 27 days	*Undaria pinnatifida*	Male KK-Ay mice (3 weeks old), fed an experimental diet with or without Fx	1,2,3	↓ TNF-α mRNA expression in WAT ↓ MCP-1 mRNA expression in WAT ↓ Blood glucose level ↑ Glucose intolerance	[145]
Meroterpenoid	90 and 180 mg MES/kg BW for 10 weeks	*Sargassum serratifolium*	Male C57BL/6J mice (6-weeks old), HCD	2	↓ MCP-1, KC concentration in serum ↓ COX-2, ICAM-1, VCAM-1, MMP-9, MCP-1, beta-actin expression in aortic tissues	[148]
Meroterpenoid	60 and 120 mg MES/kg BW/day for 8 weeks	*Sargassum serratifolium*	Male C57BL/6J mice (7-weeks old), HFD-induced obesity	2,3	↓ F4/80, MCP-1 expression in epididymal tissue ↓ Body, liver and epididymal issue weight ↓ ALT, AST ↓ TG, glucose, free fatty acid in plasma ↑ HDL cholesterol ↑ UCP-1, ADRB3 expression in subcutaneous fat	[158]
Fucoxanthin and fucoidan	275 mg LMF and 275 mg HSFx in 1 capsule, 3 capsules twice daily for 12 weeks	Unknown	Patients with nonalcoholic fatty liver disease (20–75 y/o)	3	↓ BMI ↓ ALT =liver steatosis (=CAP) =adiponectin =fasting insulin =insulin resistance	[159]

^1^ Phases of inflammation as indicated in Table 1: 1-initiation of inflammation; 2-short term amplification and regulation; 3-short- or long-term consequences; ^2^ Symbols used: ↑—increase; ↓—decrease; =—no change.
