# Supplementary material for: Brown Seaweed Food Supplementation: Effects on Allergy and Inflammation and Its Consequences
Source: Nutrients. 2021 Jul 29;13(8):2613. doi: 10.3390/nu13082613 (PMC8398742; doi:10.3390/nu13082613)
Supplement: Supplementary file 1 [file nutrients-13-02613-s001.zip › nutrients-1303544-supplementary.pdf]

SUPPLEMENTARY MATERIAL

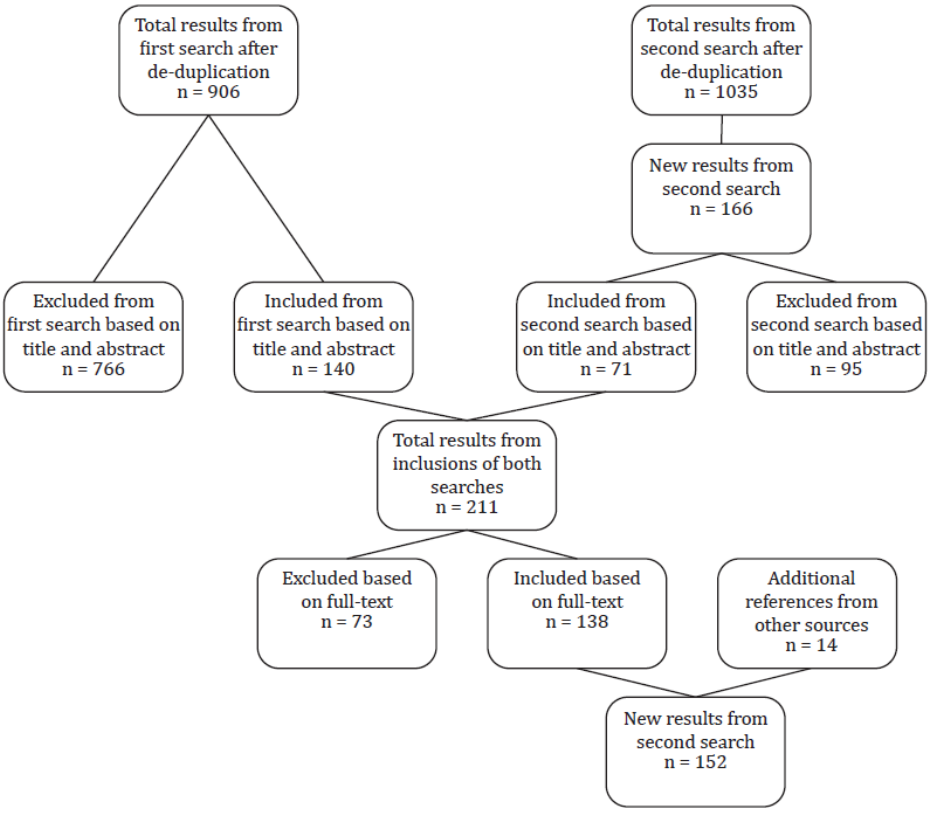

Supplementary figure S1: Flowchart of systematic search.

Search terms

Embase

('brown alga'/exp OR 'Himanthalia elongata extract'/de OR 'fucoidin'/de OR 'fucoxanthin'/de OR 'fucosterol'/de OR 'phloroglucinol'/de OR (phloroglucin* OR ascophyllum* OR ecklonia* OR laminaria* OR macrocystis* OR sargassum* OR undaria* OR saringosterol* OR fucosterin* OR fucosterol* OR fucoxanthin* OR fucoidin* OR fucoidan* OR himanthal* OR phaeophyt* OR phaeophyc* OR fucophycean* OR fucophyceae* OR oligomannate* OR phloroglucinol* OR phlorotannin* OR alaria-esculenta OR ((brown*) NEAR/3 (alga OR algae OR macroalga OR macroalgae))):ab,ti) AND ('allergy'/exp OR 'allergen'/exp OR 'leukocyte'/exp OR 'microglia'/de OR 'macrophage'/exp OR 'antigen presenting cell'/exp OR (allerg* OR atop* OR interdigitat*-cell* OR ((Langerhans OR antigen-present*) NEAR/3 (cell*)) OR histiocyt* OR kupffer* OR microglia* OR macrophage* OR dendritic-cell* OR mononuclear-cell* OR giant-cell* OR foam-cell* OR osteoclast* OR epithelioid-cell* OR monocyte* OR lymphocyte* OR leukocyt* OR leucocyt* OR granulocyt* OR basophil* OR eosinophil* OR neutrophil* OR promyelocyt* OR ((white*) NEAR/3 (cell*))):ab,ti) AND ('in vivo study'/exp OR 'follow up'/de OR 'nutraceutical'/de OR 'nutrition'/exp OR (in-vivo* OR clinic* OR case* OR cohort* OR longitud* OR prospect* OR retrospect* OR follow-up OR trial OR nutraceutic* OR nutriceutic* OR edible* OR diet* OR food* OR nutrition* OR nutrient* OR oral*):ab,ti) NOT ([Conference Abstract]/lim)

Medline
(exp Phaeophyta/ OR Phloroglucinol/ OR (phloroglucin* OR ascophyllum* OR ecklonia* OR laminaria* OR macrocystis* OR sargassum* OR undaria* OR saringosterol* OR fucosterin* OR fucosterol* OR fucoxanthin* OR fucoidin* OR fucoidan* OR himanthal* OR phaeophyt* OR phaeophyc* OR fucophycean* OR fucophyceae* OR oligomannate* OR phloroglucinol* OR phlorotannin* OR alaria-esculenta OR ((brown*) ADJ3 (alga OR algae OR macroalga OR macroalgae))).ab,ti.) AND (exp Allergens/ OR exp Leukocytes/ OR Microglia/ OR exp Macrophages/ OR exp Antigen-Presenting Cells/ OR (allerg* OR atop* OR interdigitat*-cell* OR ((Langerhans OR antigen-present*) ADJ3 (cell*)) OR histiocyt* OR kupffer* OR microglia* OR macrophage* OR dendritic-cell* OR mononuclear-cell* OR giant-cell* OR foam-cell* OR osteoclast* OR epithelioid-cell* OR monocyte* OR lymphocyte* OR leukocyt* OR leucocyt* OR granulocyt* OR basophil* OR eosinophil* OR neutrophil* OR promyelocyt* OR ((white*) ADJ3 (cell*))).ab,ti.) AND (exp Cohort Studies/ OR exp Food/ OR exp Nutrition Therapy/ OR (in-vivo* OR clinic* OR case* OR cohort* OR longitud* OR prospect* OR retrospect* OR follow-up OR trial OR nutraceutic* OR nutriceutic* OR edible* OR diet* OR food* OR nutrition* OR nutrient* OR oral*).ab,ti.) NOT (news OR congres* OR abstract* OR book* OR chapter* OR dissertation abstract*).pt.

Cochrane
((phloroglucin* OR ascophyllum* OR ecklonia* OR laminaria* OR macrocystis* OR sargassum* OR undaria* OR saringosterol* OR fucosterin* OR fucosterol* OR fucoxanthin* OR fucoidin* OR fucoidan* OR himanthal* OR phaeophyt* OR phaeophyc* OR fucophycean* OR fucophyceae* OR oligomannate* OR phloroglucinol* OR phlorotannin* OR alaria-esculenta OR ((brown*) NEAR/3 (alga OR algae OR macroalga OR macroalgae))):ab,ti) AND ((allerg* OR atop* OR (interdigitat* NEXT/1 cell*) OR ((Langerhans OR antigen-present*) NEAR/3 (cell*)) OR histiocyt* OR kupffer* OR microglia* OR macrophage* OR dendritic-cell* OR mononuclear-cell* OR giant-cell* OR foam-cell* OR osteoclast* OR epithelioid-cell* OR monocyte* OR lymphocyte* OR leukocyt* OR leucocyt* OR granulocyt* OR basophil* OR eosinophil* OR neutrophil* OR promyelocyt* OR ((white*) NEAR/3 (cell*))):ab,ti) AND ((in-vivo* OR clinic* OR case* OR cohort* OR longitud* OR prospect* OR retrospect* OR follow-up OR trial OR nutraceutic* OR nutriceutic* OR edible* OR diet* OR food* OR nutrition* OR nutrient* OR oral*):ab,ti)

Web of Science
TS=(((phloroglucin* OR ascophyllum* OR ecklonia* OR laminaria* OR macrocystis* OR sargassum* OR undaria* OR saringosterol* OR fucosterin* OR fucosterol* OR fucoxanthin* OR fucoidin* OR fucoidan* OR himanthal* OR phaeophyt* OR phaeophyc* OR fucophycean* OR fucophyceae* OR oligomannate* OR phloroglucinol* OR phlorotannin* OR alaria-esculenta OR ((brown*) NEAR/2 (alga OR algae OR macroalga OR macroalgae)))) AND ((allerg* OR atop* OR (interdigitat* NEAR/1 cell*) OR ((Langerhans OR antigen-present*) NEAR/2 (cell*)) OR histiocyt* OR kupffer* OR microglia* OR macrophage* OR dendritic-cell* OR mononuclear-cell* OR giant-cell* OR foam-cell* OR osteoclast* OR epithelioid-cell* OR monocyte* OR lymphocyte* OR leukocyt* OR leucocyt* OR granulocyt* OR basophil* OR eosinophil* OR neutrophil* OR promyelocyt* OR ((white*) NEAR/2 (cell*)))) AND ((in-vivo* OR clinic* OR case* OR cohort* OR longitud* OR prospect* OR retrospect* OR follow-up OR trial OR nutraceutic* OR nutriceutic* OR edible* OR diet* OR food* OR nutrition* OR nutrient* OR oral*))) AND DT=(Article OR Review)

Google Scholar (top 100)

phaeophyte|phaeophycean|phaeophytes|phaeophyceans|"brown alga|algae" allergy|atopic|leukocytes|lymphocytes|granulocytes|langerhans|microglia|macrophages|histiocytes|kupffer|"dendritic|giant|foam|white cells" nutraceutical|nutrition|food|diet

Glossary of Terms

increased (compared to controls)

decreased (compared to controls)

= no change (compared to controls)

AA	arachidonic acid
AAA	abdominal aortic aneurysm
ACC	acetyl-CoA carboxylase
AD	Atopic dermatitis
ADRB3	beta-3 adrenergic receptor
AE	aqueous extract
AF	Apo-9’-fucoxanthinone
AGE	advanced glycation end products
ALAT	alanine aminotransferase
ALP	alkaline phosphatase
ALT	alanine aminotransferase
ALT	alanine transaminase
ApoE	apolipoprotein E
AST	aspartate aminotransferase
AST	aspartate transaminase
BAT	brown adipose tissue
Ca	calcium
CACC	colitis associated colon cancer
CAP	controlled attenuation parameters
CAT	catalase
CCl4	carbon tetrachloride
CFA	complete Freund’s adjuvant
CNS	central nervous system
conA	concanavalin A
COX-2	cyclooxygenase 2
COX2	cyclooxygenase 2
CP	cisplatin
CRP	C-reactive protein
CY	clycophosmamide
DNC	2,4-dinitrochlorobenzene
DNCB	2,4-dinitrochlorobenzene
DNP	2,4-dinitrophenol
DNP	anti-dinitrophenyl
DSS	dextran sodium sulfate
DSS	dextran sulfate sodium
EAE	experimental autoimmune encephalomyelitis
EH	enzyme modified Sargassum fusiforme extracts
ER	Endoplasmic Reticulum (ER)
ESR	erythrocyte sedimentation rate
EtOH	ethanol
F4/80	EGF-like module-containing mucin-like hormone receptor-like 1
Fc R	Fc epsilon receptor

FD Fine dust
Fx fucoxanthin
FxOH fucoxanthinol
GFS hot water extract with galactofucan sulfate
GGT gamma glutamyl transferase
GLUT4 glucose transporter type 4
GPx glutathione peroxidase
GSH glutathione
HA hyaluronidase
Hb hemoglobin
Hc hematocrit
HCD high cholesterol diet
HDL high-density lipoprotein
HFD high fat diet
HMWF high-molecular-weight fucoidan
HOMA-IR homeostatic model assessment for insulin resistance
HSFx high stability fucoxanthin
i.n. intranasal
i.p. intraperitoneal
ICAM-1 intracellular adhesion molecule 1
ICR Institute of Cancer Research
IFA incomplete Freund's adjuvant
IFN-ɣ Interferon gamma
Ig immunoglobulin
iNOS inducible nitric oxide
KC keratinocyte chemoattractant
LBP lipopolysaccharide-binding protein
LD Borrelia burgdorferi
LDH lactate dehydrogenase
LDL low-density lipoprotein
LDL low-density lipoprotein
LFD low-fat diet
LMF low-molecular-weight
LMWF low-molecular-weight fucoidan
LOX lipoxygenase
LPS lipopolysaccharide
LXR liver X receptor
M1 pro-inflammatory macrophage (classically activated)
M2 anti-inflammatory macrophage (alternatively activated)
MAPK mitogen-activated protein kinase
MBP Myelin basic protein
MCP-1 monocyte chemotactic protein-1
MDA malondialdehyde
mDC myeloid dendritic cell
MeOH methanol
MES meroterpenoid-rich fraction of ethanolic extract from *Sargassum serratifolium*
MetS metabolic syndrome
MIP-2 macrophage inflammatory protein 2
MLN mesenteric lymph node
MMP-9 matrix metallopeptidase 9
MMWF medium-molecular-weight fucoidan

MPO myeloperoxidase
NAFLD nonalcoholic fatty liver disease
NF-κB nuclear factor-κB
NH Sargassum fusiforme extracts
NK natural killer cell
NO nitric oxide
OVA ovalbumin
OXA oxazolone
oxLDL oxidized low-density lipoprotein
PCA passive cutaneous anaphylaxis
PCON polycystic ovary syndrome
PCOS polycystic ovary syndrome
pDC plasmacytoid dendritic cell
PFF phlorofucofuroeckol
PGC1α peroxisome proliferator-activated receptor gamma coactivator 1-alpha
PGE2 prostaglandin E2
PHB 2,7-phloroglucinol-6,6-bieckol
PI-3K phosphatidyl inositol-3 kinase
PLA2 phospholipase A2
PM particulate matter
PMN polymorphonuclear leukocytes
PON-1 serum paraoxonase-1
PPAR peroxisome proliferator-activated receptor
PPB pyrogallol-phloroglucinol-6,6’-bieckol
PREC phlorotannin-rich extract of Ecklonia cava
QOL quality of life
RAGE receptor for advanced glycation end-products
RBC red blood cells
ROS reactive oxygen species
SDF-1 stromal cell-derived factor 1
SIRT1 sirtuin 1
SMCs smooth muscle cells?
SOCS-3 suppressor of cytokine signaling 3
SOD superoxide dismutase
TG triglyceride
TIMP-1 tissue inhibitor of metalloproteinase 1
TPA 12-O-tetradecanoylphorbol-13-acetate
UCP-1 uncoupling protein 1
VCAM-1 vascular cell adhesion molecule 1
WAT white adipose tissue
WBC white blood cells
WL wakame lipids
WSSV White Spot Syndrome Virus
